# The Major Peanut Allergen Ara h 2 Produced in *Nicotiana benthamiana* Contains Hydroxyprolines and Is a Viable Alternative to the *E. Coli* Product in Allergy Diagnosis

**DOI:** 10.3389/fpls.2021.723363

**Published:** 2021-10-04

**Authors:** Öykü Üzülmez, Tanja Kalic, Vanessa Mayr, Nina Lengger, Angelika Tscheppe, Christian Radauer, Christine Hafner, Wolfgang Hemmer, Heimo Breiteneder

**Affiliations:** ^1^Institute of Pathophysiology and Allergy Research, Medical University of Vienna, Vienna, Austria; ^2^Department of Dermatology, University Hospital St. Pölten, Karl Landsteiner University of Health Sciences, St. Pölten, Austria; ^3^Karl Landsteiner Institute for Dermatological Research, St. Pölten, Austria; ^4^FAZ - Floridsdorf Allergy Center, Vienna, Austria

**Keywords:** peanut allergy, *Nicotiana benthamiana*, Ara h 2, transient expression, PTM, hydroxyproline

## Abstract

Peanut allergy is a potentially life-threatening disease that is mediated by allergen-specific immunoglobulin E (IgE) antibodies. The major peanut allergen Ara h 2, a 2S albumin seed storage protein, is one of the most dangerous and potent plant allergens. Ara h 2 is posttranslationally modified to harbor four disulfide bridges and three hydroxyprolines. These hydroxyproline residues are required for optimal IgE-binding to the DPYSP^OH^S motifs representing an immunodominant IgE epitope. So far, recombinant Ara h 2 has been produced in *Escherichia coli, Lactococcus lactis, Trichoplusia ni* insect cell, and *Chlamydomonas reinhardtii* chloroplast expression systems, which were all incapable of proline hydroxylation. However, molecular diagnosis of peanut allergy is performed using either natural or *E. coli*-produced major peanut allergens. As IgE from the majority of patients is directed to Ara h 2, it is of great importance that the recombinant Ara h 2 harbors all of its eukaryotic posttranslational modifications. We produced hydroxyproline-containing and correctly folded Ara h 2 in the endoplasmic reticulum of leaf cells of *Nicotiana benthamiana* plants, using the plant virus-based magnICON^®^ transient expression system with a yield of 200 mg/kg fresh biomass. To compare prokaryotic with eukaryotic expression methods, Ara h 2 was expressed in *E. coli* together with the disulfide-bond isomerase DsbC and thus harbored disulfide bridges but no hydroxyprolines. The recombinant allergens from *N. benthamiana* and *E. coli* were characterized and compared to the natural Ara h 2 isolated from roasted peanuts. Natural Ara h 2 outperformed both recombinant proteins in IgE-binding and activation of basophils *via* IgE cross-linking, the latter indicating the potency of the allergen. Interestingly, significantly more efficient IgE cross-linking by the *N. benthamiana*-produced allergen was observed in comparison to the one induced by the *E. coli* product. Ara h 2 from *N. benthamiana* plants displayed a higher similarity to the natural allergen in terms of basophil activation due to the presence of hydroxyproline residues, supporting so far published data on their contribution to the immunodominant IgE epitope. Our study advocates the use of *N. benthamiana* plants instead of prokaryotic expression hosts for the production of the major peanut allergen Ara h 2.

## Introduction

Type 1 allergy, an immune disorder mediated by immunoglobulin E (IgE) antibodies, affects up to 30% of the world population (Valenta et al., 2018a). Allergy to peanut is particularly important as it is one of the most persistent types of allergies, is rarely outgrown, and may lead to life-threatening reactions such as anaphylaxis (Chinthrajah et al., 2020). In developed countries, the incidence of peanut allergy has been increasing due to the enhanced exposure to and consumption of peanuts (Platts-Mills, 2015; Reynolds and Finlay, 2017). The seeds of the peanut plant (*Arachis hypogaea*) harbor 18 different allergenic proteins (http://www.allergen.org/), including the highly potent 2S albumin Ara h 2, which makes up 6–9% (1.6–2.3 g/kg) of the total seed protein (Koppelman et al., 2001). Besides their biological role as defensive proteins to protect the seeds (Ozias-Akins and Breiteneder, 2019), seed storage proteins are crucial sources of nutrients for the growing seedling (Kang et al., 2007). Ara h 2 has a 21 amino acid residue N-terminal signal peptide, which directs the protein to the seed storage vacuoles and is then cleaved off, yielding the mature protein (Li et al., 2010). 2S albumins are a member family of the prolamin superfamily, which contains the highest number of allergens of any protein superfamily (Radauer et al., 2008). Peanuts express three allergenic 2S albumins, namely Ara h 2, Ara h 6, and Ara h 7 (http://www.allergen.org/search.php?Species=Arachis%hypogaea). Ara h 7 has been recently described to have partial cross-reactivity with Ara h 2 (Apostolovic et al., 2021). Co-sensitization to Ara h 2 and Ara h 6 is common for peanut allergic patients, as these allergens share 60% sequence identity (Hemmings et al., 2020). Yet, Ara h 2 demonstrates a superior accuracy for the diagnosis of peanut allergy, since over 90% of peanut-sensitized patients develop IgE antibodies to it (Zhuang and Dreskin, 2013). Ara h 2 occurs in four isoforms of 16–18 kDa. The longer isoforms Ara h 2.0201 and Ara h 2.0202 vary by one residue. They differ from the shorter isoforms Ara h 2.0101 and Ara h 2.0102 by an additional repetition of the DPYSPS motif, which is an immunodominant IgE epitope (Stanley et al., 1997; Li et al., 2010). Ara h 2 harbors two posttranslational modifications (PTMs), namely, four disulfide bonds and three hydroxyprolines, the latter being located within the repeated DPYSPS immunodominant epitopes (Li et al., 2010). Although natural Ara h 2 has a canonical N-glycosylation site (NQS), it is not glycosylated (Li et al., 2010).

Current *in vivo* diagnosis of allergies is mainly performed with natural extracts of allergen sources, employing skin tests or oral/nasal challenges (Üzülmez et al., 2020). However, it is notable that the allergen contents in extracts vary by manufacturer and that most extracts are not standardized (Curin et al., 2017). Most importantly, some natural allergens cannot be administered *in vivo* due to their high potency to induce symptoms. Hence, *in vitro* tests with purified natural (n) or recombinant (r) allergens are preferable for at-risk patients. Isolated natural allergens have been largely replaced by recombinant allergens produced in prokaryotic hosts, mainly in *Escherichia coli* (Larsen et al., 2020) due to easier production, simpler characterization and standardization of the products, and the absence of cross-contamination with other allergens from the same source, which could influence the test results (Lowenstein and Larsen, 2001; Sancho et al., 2010; Curin et al., 2017). *Escherichia coli* and *Lactococcus lactis* have been utilized to produce rAra h 2 with low-to-moderate IgE-binding capacities (Astier et al., 2006; Lew and Lim, 2016; Chan et al., 2020). IgE from all peanut allergic patients involved in the study recognized the *E. coli*-made rAra h 2; thus, authors reported this recombinant allergen as a promising candidate for its use in allergy diagnosis (Astier et al., 2006). Although tested with sera from peanut allergic mice, IgE-binding of natural Ara h 2 did not differ from those made in *E. coli* or *L. lactis* (Chan et al., 2020). Another study reported significantly weaker recognition of the *E. coli*-made rAra h 2 by IgE of allergic patients in comparison to the natural protein (Lew and Lim, 2016). To our knowledge, no plant-produced recombinant allergen is used in any of the commercially available singleplex or multiplex diagnostic assays. Molecular allergy diagnosis of peanut allergy is performed with either natural or *E. coli*-produced rAra h 2 in the singleplex ImmunoCAP (r) assay (Park et al., 2018) and in multiplex assays, such as the ImmunoCAP ISAC (r) (Gadisseur et al., 2011), the experimental MeDALL-chip (n) (Lupinek et al., 2014), or the ALEX allergy explorer (r) (Hoang et al., 2020).

Eukaryotic recombinant expression systems are favorable for producing recombinant allergens with their natural PTMs to be used in allergy diagnosis and for providing templates for designing hypoallergens for allergen immunotherapy. Obtaining naturally occurring PTMs during recombinant protein expression is of great importance as they are critical contributors to the IgE-binding of allergens (Petersen et al., 1998; Bernard et al., 2000, 2015; Bublin et al., 2003; Schmid-Grendelmeier et al., 2003; Barral et al., 2005; Leonard et al., 2005; Halim et al., 2015). Eukaryotic systems, such as chloroplasts of the unicellular alga *Chlamydomonas reinhardtii* and *Trichoplusia ni* insect cells, have been used for the recombinant production of Ara h 2 (Gregory et al., 2016; Tscheppe et al., 2020). Although, from eukaryotic hosts, both the green alga- and the insect cell-made rAra h 2 fell short of the performance of the natural allergen in IgE-binding assays (Gregory et al., 2016; Tscheppe et al., 2020). Gregory et al. suggested that distorted conformational epitopes and possible acetylation in chloroplasts were the reasons for weaker IgE-binding of algal-produced peanut allergens in comparison to their native counterparts (Gregory et al., 2016). When produced in insect cells, the disulfide bond formation was achieved (Tscheppe et al., 2020), yet there has been no report of rAra h 2 production with hydroxyprolines until now. The importance of both of its PTMs, namely the disulfide bonds and the hydroxyprolines, for the IgE-reactivity of Ara h 2 has been reported (Bernard et al., 2015; Tscheppe et al., 2020). When the cysteine residues were reduced and alkylated to permanently destroy the natural fold of the protein, the IgE-binding and anaphylaxis-inducing capacity of Ara h 2 decreased notably (Tscheppe et al., 2020). Moreover, hydroxyproline-containing Ara h 2 peptides had a 1,000-fold higher affinity to IgE in comparison to the peptides without this modification (Bernard et al., 2015).

Recombinant expression of allergens in plant systems offers the advantages of utilizing the eukaryotic translation machinery to add PTMs, human pathogen-free production, safe *in vivo* application, and safe use for oral tolerance induction by bioencapsulation (Schmidt et al., 2008; Daniell et al., 2019). Plant virus genomes have been engineered for the production of proteins-of-interest by taking advantage of the compromised viral defense mechanisms of *Nicotiana benthamiana* plants (Yang et al., 2004). The application of plant virus-based vectors has resulted in the production of several plant-made pharmaceuticals that are either in clinical trials or already approved (Gleba et al., 2014; Giritch et al., 2017). While it takes several months to generate a fully transgenic offspring *via* stable transformation (Slater et al., 2008), transient systems offer scalable, quick, systemic/local infection, utilizing RNA virus-based vector systems such as magnICON® (Marillonnet et al., 2005), TRBO (Lindbo, 2007), and pEAQ (Lomonossoff and D'Aoust, 2016) or DNA virus-based one, such as geminivirus (Yamamoto et al., 2018), allowing high-yield production of recombinant proteins within 1–2 weeks. Moreover, typical yields for stably transformed plants are below 100 mg/kg (Schillberg et al., 2019), whereas transient expression yields can be scaled up to 5 g/kg (Marillonnet et al., 2004).

*N. benthamiana* has been utilized for the transient cytosolic expression of allergens from birch pollen (Bet v 1) and latex (Hev b 1, Hev b 3) by inoculating plant leaves with *in vitro* transcribed viral RNA, which were all shown to bind IgE from allergic patients (Krebitz et al., 2000; Breiteneder et al., 2001). Later, viral vectors were utilized for the transient expression of several plant allergens such as Mal d 2 (apple), Bet v 1 (birch), and Gly m 8 (soybean), which were able to bind allergen-specific IgE (sIgE) from the sera of allergic patients or monoclonal antibodies (Krebitz et al., 2003; Santoni et al., 2019; Ueberham et al., 2019; Yamada et al., 2020). Yamada et al. reported the highest yield ever achieved for a recombinant allergen produced in plants for Bet v 1 (1.2 g/kg fresh biomass), using a deconstructed geminivirus vector system. Yet an undesired glycosylation of the allergen was observed due to the canonical N-glycosylation site within the Bet v 1 sequence (Yamada et al., 2020). Santoni et al. (2019) reported that sIgE from sera of allergic patients directed to plant-produced Bet v 1 was significantly lower than the one directed to the recombinant protein from *E. coli*, which was attributed to the misfolding of the *N. benthamiana*-produced allergen.

Our study describes the detailed characterization of *Agrobacterium*-driven transient expression of rAra h 2 in the leaves of *N. benthamiana*. We employed the tobacco mosaic virus (TMV)-based magnICON^®^ expression vectors (Klimyuk et al., 2014) for the production of hydroxyproline-containing and correctly folded Ara h 2. We performed a detailed biochemical and immunological characterization of plant-produced rAra h 2 and investigated the impact of hydroxyproline residues on allergenicity by comparing recombinants from *N. benthamiana* and *E. coli* with natural Ara h 2.

## Materials and Methods

### Construction of the Tobacco Mosaic Virus (TMV)-Based Expression Provectors

The TMV-based expression provectors pICH31070, pICH20155, and pICH14011 were supplied by Dr. Victor Klimyuk of ICON Genetics (Halle, Germany; since 2017, fully owned by Denka Co. Ltd., Tokyo, Japan). A combination of plasmids encoding transgenes framed by left and right T-DNA borders (LB and RB, respectively) was used for *Agrobacterium*-mediated heterologous protein expression in *N. benthamiana* plants. This modular system comprises (i) a 5′-provector (pICH20155) encoding viral genes necessary for plant transformation, (ii) a 3′-provector encoding the gene-of-interest (pICH31070), (iii) a separate plasmid encoding the PhiC31 integrase (pICH14011) that enables *in planta* recombination of both modules (Marillonnet et al., 2004).

The 5′-provector pICH20155, as described before (Kalthoff et al., 2010), encodes the viral movement protein (MP), the viral RNA-dependent RNA polymerase (RdRP) and the rice α-amylase endoplasmic reticulum (ER)-targeting peptide (SP). The 3′-provector pICH31070, which was described by Engler et al. (2008), encodes the gene-of-interest. The third TMV-based plasmid pICH14011, described elsewhere (Kalthoff et al., 2010), encodes the PhiC31 integrase, which then fused the ER-targeting peptide in-frame with the gene-of-interest as a result of the ligation of the 5′- and 3′-modules. The 5′ ER-targeting peptide present in the pre-protein is then cleaved off during protein maturation in the ER (Chen et al., 2004). The sequence of mature Ara h 2.0201 (Uniprot Q6PSU2, aa 22-172), including a single amino acid substitution at N106 to Q and a C-terminal hexa-histidine tag, followed by an ER-retention signal (SEKDEL), was codon-optimized for *N. benthamiana* and synthesized and ligated into the pEX-A128 cloning vector (Eurofins Genomics, Austria). The subcloning was performed *via* BsaI restriction sites, following the Golden Gate cloning protocol (Engler et al., 2008). The resulting plasmid was referred to as pICH31070_Ara h 2 N106Q SEKDEL.

### Agroinfiltration of *N. benthamiana* Plants and Protein Expression

Electrocompetent *Agrobacterium tumefaciens* (GV3101) cells (recently renamed to *Rhizobium radiobacter*) were transformed with either pICH14011, pICH20155, or pICH31070. The transformed bacteria were grown in YEB medium (0.1% yeast extract, 0.5% beef extract, 0.5% peptone, 0.5% sucrose, 0.05% MgSO_4_.7H_2_O, pH 7.0) with the antibiotics kanamycin (50 mg/L) and rifampicin (25 mg/L) to an OD_600_ of 1.5–2 at 28°C. The cultures were then centrifuged at 8,500 × *g* for 1 h at 4°C. The pellets were resuspended in infiltration buffer (10 mM MES, 10 mM MgSO_4_, 2% sucrose, 100 μM acetosyringone, pH 5.5), and the OD_600_ was adjusted to 1.0 by mixing three transformants in a 1:1:1 ratio. The leaves of 7-week-old *N. benthamiana* plants (grown at 24°C with 16 h light, 8 h dark) were infiltrated using a syringe without a needle with the mixture of agrobacteria suspensions, carrying either of the three plasmids.

### Protein Extraction From *N. benthamiana* Leaves

Infected leaves were collected 10 days post-infiltration, snap frozen in liquid nitrogen and ground using a mortar and a pestle. One gram of ground-up leaves was resuspended in 3 mL of the extraction buffer (50 mM Tris, 150 mM NaCl, 1 mM DTT, 0.1% Triton X-100, pH 7.5), containing a cocktail of protease inhibitors (Cat. No. 11873580001, Roche, Basel, Switzerland). Proteins were extracted by vigorously vortexing the ground-leaf suspensions at 4°C. Additional incubation of the total leaf suspensions overnight at −20°C helped with cell lysis. Thawed suspensions were cleared by centrifugation at 14,000 × *g* for 30 min at 4°C. Total soluble and insoluble fractions containing rAra h 2 were prepared as follows. An aliquot from the homogenized leaf suspension was taken for mixing with the reducing sample buffer (200 mM Tris, 300 mM DTT, 4% SDS, 40% glycerine, bromphenol blue), heated at 95°C and loaded as the total fraction. The rest of the homogenized leaf suspension was centrifuged at 14,000 × *g* for 15 min. An aliquot from the supernatant was mixed with the reducing sample buffer, heated at 95°C and loaded as the soluble fraction. The pellet was mixed with the reducing sample buffer, and an aliquot was taken for heating at 95°C prior to loading.

### Protein Purification and Analysis by SDS-PAGE and Western Blotting

All chromatographic steps were performed using an FPLC system. The supernatant of the crude extract was dialyzed against the loading buffer (25 mM Tris, 3 M NaCl, 10 mM imidazole, pH 8.0). Filtered samples were applied to a Ni-NTA-agarose column (Cat. No. 1018244, Qiagen, Netherlands), and rAra h 2 was eluted by adding 25 mM to 100 mM imidazole. As a second purification step, anion exchange chromatography was performed using a MonoQ column (Cat. No. 17-5166-01, GE Healthcare, Illinois, USA). rAra h 2 was loaded onto the column in buffer A (20 mM Tris, pH 8.0) and eluted with increasing NaCl concentrations, recovering the protein at around 220 mM. Pure proteins were concentrated and dialyzed against phosphate-buffered saline (PBS) (10 mM Na_2_HPO_4_, 136 mM NaCl, 2.7 mM KCl, 1.8 mM KH_2_PO_4_, pH 7.4) before measuring the concentration using the bicinchoninic acid assay (BCA) (Cat. No. 23225, Thermo Fisher Scientific, Massachusetts, USA).

Two micrograms of purified protein per lane were run on an SDS-PAGE. Samples were mixed with the 4× sample buffer either with or without DTT for reducing and non-reducing conditions, respectively. Protein samples were heated at 95°C for 10 min prior to loading onto the gel. Gels were run at 120 V for 40 min and stained with Coomassie Brilliant Blue R-250 (CBB). For Western blot analysis, unstained gels were blotted onto nitrocellulose membranes (Cat. No. 10600007, GE Healthcare, Illinois, USA) at 400 mA constant for 1 h at room temperature. Membranes were incubated with the anti-Ara h 2 monoclonal antibody 1C4 (Cat. No. EL-AH2, Indoor Biotechnologies, Charlottesville, Virginia, USA) (1:1,000) or with sera from patients (1:10). An anti-mouse IgG + IgM conjugated to alkaline phosphatase (AP) (Cat. No. 315-055-048, Dianova GmbH, Hamburg, Germany) (1:5,000) or anti-human IgE conjugated to AP (Cat. No. 555859, BD Pharmingen, California, USA) were used as secondary antibodies, respectively. 5-Bromo-4-chloro-3-indoyl phosphate (BCIP) and nitro blue tetrazolium chloride (NBT) were used for developing the signals. For percentage of soluble protein analysis, the immunoblot was analyzed using ImageJ 1.8.0 (Schneider et al., 2012).

### Purification of nAra h 2 From Peanut Kernels

nAra h 2 was extracted from roasted peanuts, bought from a local supermarket. Desalted peanuts were ground to a fine powder. Following defatting with hexane, proteins were extracted with H_2_O, and the particulate matter was removed *via* centrifugation at 18,900 × *g* for 2 h at 4°C. nAra h 2 was isolated by anion exchange and hydrophobic interaction chromatography as previously described (Palladino et al., 2018).

### rAra h 2 Expression in *E. coli* SHuffle T7 Express lysY Cells

The wild-type sequence of Ara h 2.0201 was subcloned into the pMWHisN expression vector, an in-house designed T7 expression vector based on pMW172 (Way et al., 1990) that encoded an eight amino acid-long extension, including a hexa-histidine tag fused to the N-terminus of the inserted sequence. The expression was performed in 2 × TY medium (1.6% peptone, 1% yeast extract, 0.5% NaCl, pH 7.4), supplemented with ampicillin (100 μg/mL) at 30°C for 10 h. The expression was induced using 0.4 mM IPTG. Bacterial pellets were resuspended in the sodium phosphate buffer (9.7 mM NaH_2_PO_4_, 40.3 mM Na_2_HPO_4_, pH 7.5) with 5 mM imidazole and an EDTA-free protease inhibitor cocktail (Cat. No. 04693132001, Roche, Switzerland) and lysed three times by high-pressure cell disruption. Lysed pellets were centrifuged at 18,000 × *g* for 1 h at 4°C. To remove the bacterial DNA, the supernatant was treated with 1:1,000 Biocryl BPA-1,000 (Tosoh Bioscience, Griesheim, Germany). Histidine-affinity chromatography purification was performed as explained before, and rAra h 2 was recovered at around 50 mM imidazole. Anion-exchange chromatography was performed as explained before, recovering the protein at around 170 mM NaCl. Pure proteins were concentrated and dialyzed against PBS before assessing the concentration using the BCA (Cat. No. 23225, Thermo Fisher Scientific, Waltham, Massachusetts, USA).

### Mass Spectrometry

For confirming the sequence and PTMs, rAra h 2 expressed in *N. benthamiana* was run under reducing conditions on an SDS-PAGE, visualized by CBB staining, and the corresponding band was excised from the gel. Mass spectrometry analyses were then performed by the Mass Spectrometry Facility at Max Perutz Labs using the instrument pool of the Vienna BioCenter Core Facilities. The gel samples were processed as previously described (Mair et al., 2015). Briefly, gel pieces were cut and washed in an ammonium bicarbonate buffer. Disulfide bridges were reduced with dithiothreitol and alkylated with iodoacetamide. Following tryptic digestion, peptides were extracted from the gel by sonication. Peptide samples were separated on an Ultimate 3000 RSLC nano-flow chromatography system and analyzed on a Q Exactive HF Orbitrap mass spectrometer (both Thermo Fisher Scientific, Waltham, Massachusetts, USA), running a data-dependent acquisition method (Frohner et al., 2020). Raw data were interpreted with MaxQuant 1.6.0.16 (Cox and Mann, 2008), searching against the databases of *N. benthamiana*, TMV, contaminants, and rAra h 2 sequences with tryptic specificity. Hydroxylation of prolines and oxidation of methionine, HexNAc attachments on serine or threonine, and N-terminal protein acetylation were set as variable modifications, and carbamidomethylation of cysteines as fixed modification. The data were filtered at a 1% false discovery rate at the level of peptide-spectrum matches and at a protein level. The spectra were manually searched for oxonium ions. Hydroxyproline-containing peptides were manually inspected.

### Characteristics of Peanut-Allergic Patients

Twenty adults (aged 18–54 years) with a medical history of peanut allergy were recruited at the Department of Dermatology, University Hospital St. Pölten (Supplementarey Table 1). Sera from patients were separated *via* centrifugation at 1,800 × *g* for 10 min and stored at −20°C until use. Total IgE, sIgE to peanut extract, and to individual peanut allergens (Ara h 1, Ara h 2, Ara h 3, Ara h 8, and Ara h 9) were measured by ImmunoCAP (Thermo Fisher Scientific, Waltham, Massachusetts, USA). A value higher than 0.35 kUA/L indicated a positive result. Skin-prick tests were performed with commercial peanut extracts, and results were considered positive when the wheal diameter was ≥3 mm compared with the saline control. Three non-atopic controls (P21, P22, and P23) without a history of type 1 allergy, and three atopic patients (P24, P25, and P26) with allergies other than peanut were recruited as controls (Supplementary Table 1). Power calculations of sample size for comparing IgE-reactivities of the three Ara h 2 proteins were done using the G^*^Power 3.1.9.4 software. The sample size was found sufficient to detect differences between groups of about 0.6 standard deviation with a power of 90%.

### Determination of IgE-Binding Activity of Ara h 2 Proteins by ELISA and RBL Assay

For ELISA, pure proteins were coated onto microtiter plates overnight at 4°C at 2 μg/mL protein in sodium carbonate buffer (50 mM NaHCO_3_, pH 9.5). After blocking with 3% skimmed milk powder in Tris-buffered saline (TBS) (50 mM Tris, 150 mM NaCl at pH 7.4) with 0.5% Tween-20, wells were incubated overnight at 4°C with sera from 20 patients with peanut allergy, three non-atopic patients, or two atopic patients without peanut allergy (diluted 1:10). An anti-human IgE conjugated to AP (Cat. No. 555859, BD Biosciences, San Jose, California, USA) was used for detection, and the reaction was developed using *p*-nitrophenyl phosphate tablets (Cat. No. N2770, Sigma-Aldrich Merck, Germany). The plates were measured after 20 min at OD_405_ (Tecan microplate reader, Tecan, Männedorf, Switzerland), using the SparkControl Magellan 2.2 software. The plotted values were calculated by subtracting the mean value obtained from all negative controls plus three times the standard deviation from the raw data.

Basophil activation test (BAT) using human IgE-receptor expressing rat basophil leukemia cells (RBL) were performed following a published protocol (Nakamura et al., 2010). All sera were diluted 1:20, and cells were sensitized overnight. After 17 h, allergen stimulation was performed at various concentrations. Luciferase substrate was added, and the resulting luminescence was measured (Tecan microplate reader). Data were converted to stimulation indices (SI) by dividing the triplicate mean value of treated cells by the value of non-stimulated cells. The threshold for positive values was set to two.

### Statistical Analysis

The Friedman test with the Dunn posttest was performed to compare IgE-binding and basophil activating performance of different proteins. The results from the ELISA and RBL assays were correlated with the Ara h 2-ImmunoCAP values of the patients by computing Spearman's rank correlation coefficients (*r*) and two-tailored *P*-values. All analyses were done using GraphPad Prism 9.0.0 (GraphPad Software, California, USA).

## Results

### Production and Purification of Recombinant and Natural Ara h 2 Proteins

**Figure 1:**
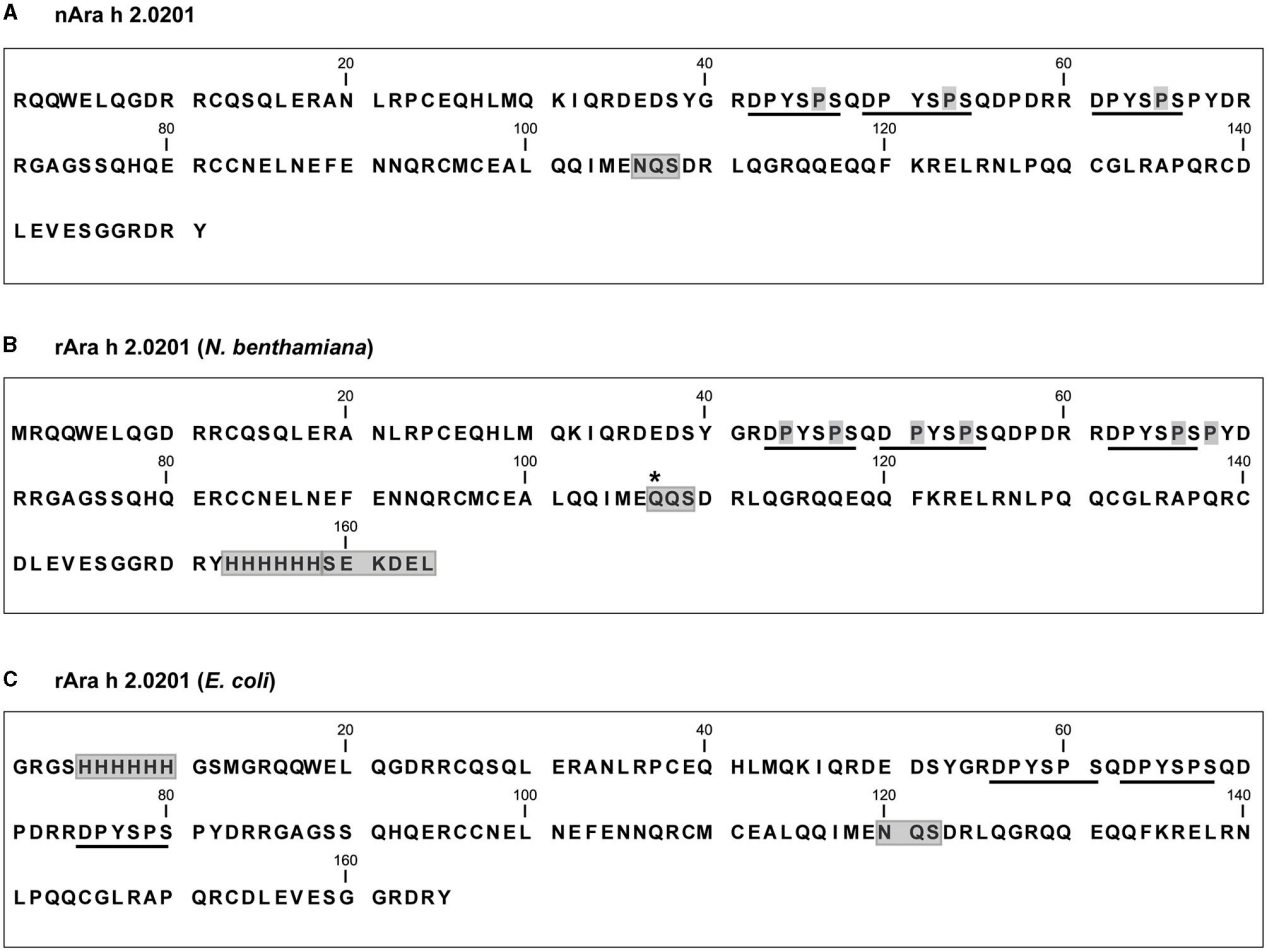
Amino acid sequences of the Ara h 2 proteins used in this study. **(A)** Mature nAra h 2.0201. Naturally occurring hydroxyproline residues within the immunodominant IgE epitopes (underlined) are highlighted in gray. The canonical N-glycosylation site (NQS) is boxed. **(B)** rAra h 2.0201 used for expression in *N. benthamiana*, including a C-terminal hexa-histidine tag and an ER retention signal (SEKDEL, boxed). The canonical N-glycosylation site NQS was mutated to QQS (boxed), and the single amino acid substitution is marked with an asterisk. Mass spectrometry-detected and manually confirmed hydroxyproline residues are highlighted in gray. **(C)** rAra h 2.0201expressed in *E. coli*, including an N-terminal hexa-histidine tag (boxed). The canonical N-glycosylation site is boxed.

An undesired glycosylation due to the presence of a canonical N-glycosylation site was reported for Bet v 1 when expressed in *N. benthamiana* leaves (Yamada et al., 2020). Ara h 2 also contains a canonical N-glycosylation site within its sequence (Figure 1A). Hence, for the expression of rAra h 2 in *N. benthamiana* leaves, we designed a construct based on the longer isoform, Ara h 2.0201, with a single amino acid substitution (N106Q) to alter the canonical N-glycosylation site (Figure 1B). The rice α-amylase ER-targeting peptide encoded by the 5′-pICH20155 plasmid is ligated in-frame to the N-terminal part of Ara h 2 encoded by the 3′-provector, yet after translocation into the ER, the signal peptide is cleaved off and thus is not present in the mature protein (Chen et al., 2004). Moreover, a C-terminal hexa-histidine tag was added followed by an ER retention signal (SEKDEL), which prevented Golgi-mediated O-linked glycosylation (Schoberer et al., 2018) (PRIDE repository dataset identifier: PXD027015). The tobacco codon-optimized sequence encoding Ara h 2 N106Q SEKDEL was amplified and subcloned within the left and right T-DNA borders of the 3'-pICH31070 plasmid (Supplementary Figure 1A). A successful infection of *N. benthamiana* leaves was achieved by *in planta* recombination of the 5'-provector pICH20155 carrying viral genes with the 3'-provector pICH31070 encoding the Ara h 2 sequence.

Crude extracts from infiltrated and sham-treated *N. benthamiana* leaves were harvested 10 days post-infiltration and prepared by dissolving ground-up leaves in the extraction buffer and analyzed for Ara h 2 expression by SDS-PAGE (Supplementary Figure 1B). The concentration of rAra h 2 was measured by the BCA, following the purification *via* immobilized metal affinity chromatography. The magnICON^®^ system yielded 200 mg of rAra h 2 per kg fresh biomass (Table 1). Homogenized leaf suspensions of *N. benthamiana* were analyzed by immunoblotting using a monoclonal anti-Ara h 2 antibody (Supplementary Figure 1C). The soluble fraction of the homogenized leaf suspensions contained approximately 40% of the total expressed Ara h 2 (Supplementary Figure 1D).

**Table 1:** Protein characteristics of the Ara h 2 proteins used in this study.

**ID**	**Produced in**	**PTMs**		**Mw (Da)**	**Length (residues)**	**Yield**
		**Hydroxyproline positions**	**S-S**			
nAra h 2.0201/2 and nAra h 2.0101/2	*Arachis hypogaea*	Yes at P46, P53, P65	Yes	17,994 and 16,047	151 and 135	1.6–2.3 g/kg of peanuts (Koppelman et al., 2001)
rAra h 2.0201 N106Q SEKDEL	*Nicotiana benthamiana*	Yes at P43, P46, P50, P53, P65, P67	Yes	19,663	164	200 mg/kg fresh biomass
rAra h 2.0201	*Escherichia coli*	No	Yes	19,506	165	0.3 mg/L liquid culture

*Hydroxyproline residues of N. benthamiana-produced rAra h 2 are numbered, excluding the N-terminal methionine*.

The longer isoform Ara h 2.0201, including an N-terminal hexa-histidine tag, was expressed in the Shuffle^®^ Express LysY strain of *E. coli*, which provided disulfide bridge formation due to the constitutive expression of the disulfide-bond isomerase DsbC (Figure 1C, Table 1). rAra h 2 from *E. coli* was purified using metal affinity chromatography, and the concentration was measured by the BCA. To compare the bioequivalence of rAra h 2 proteins produced in *N. benthamiana* and *E. coli* with the natural allergen, isoforms Ara h 2.0101/2 and Ara h 2.0201/2 were extracted from roasted peanuts (Table 1).

### Comparison of the Biochemical Properties of rAra h 2 Expressed in *N. benthamiana* With nAra h 2 and rAra h 2 Expressed in *E. coli*

**Figure 2:**
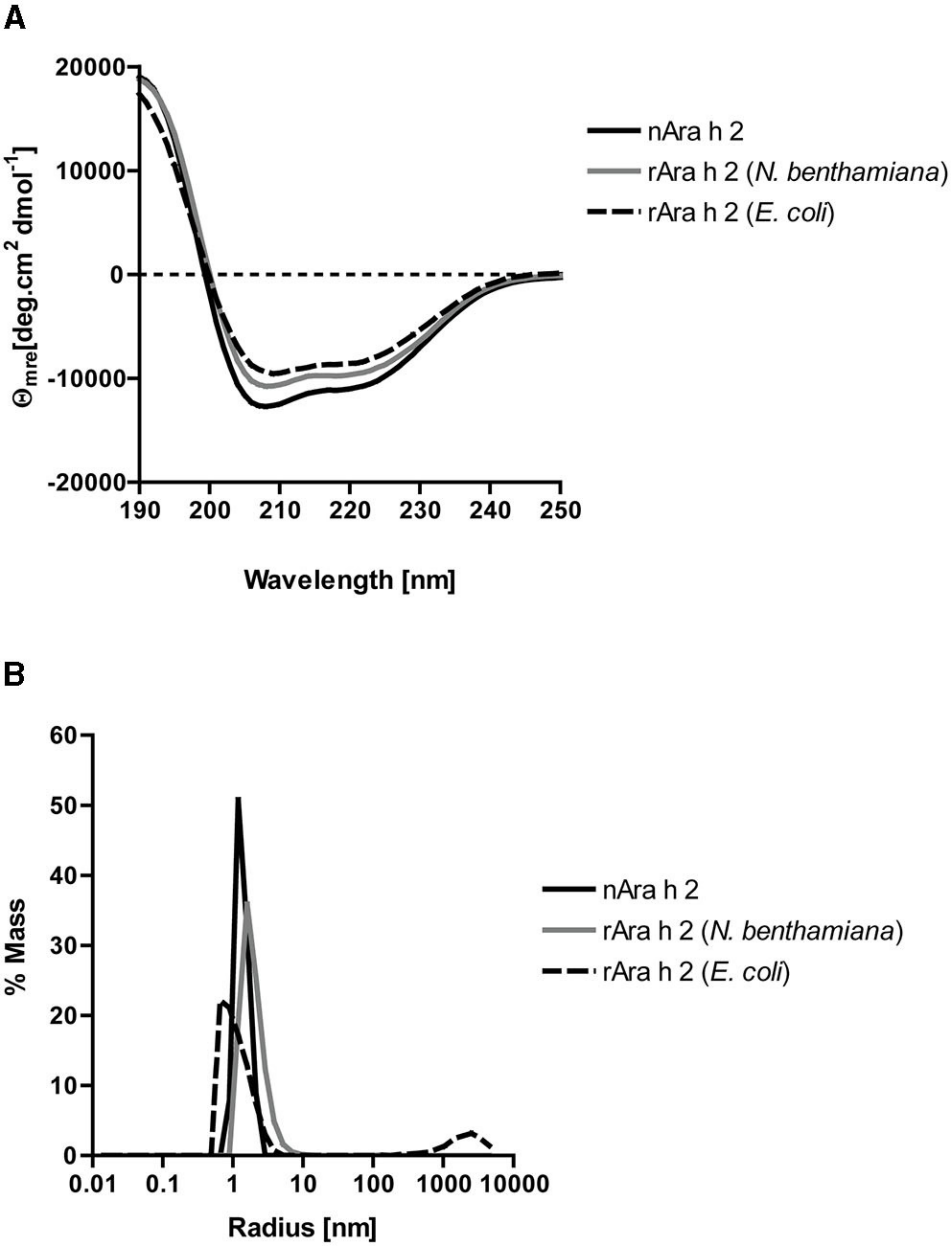
Biochemical characterization of nAra h 2, rAra h 2 (*N. benthamiana*), and rAra h 2 (*E. coli)*. **(A)** Circular dichroism spectra showing double minima at 208 and 222 nm indicated exclusively alpha helical content for all three proteins. **(B)** Dynamic light scattering measured monomeric states for all three proteins, except for rAra h 2 from *E. coli*, which contained 1% aggregates.

**Figure 3:**
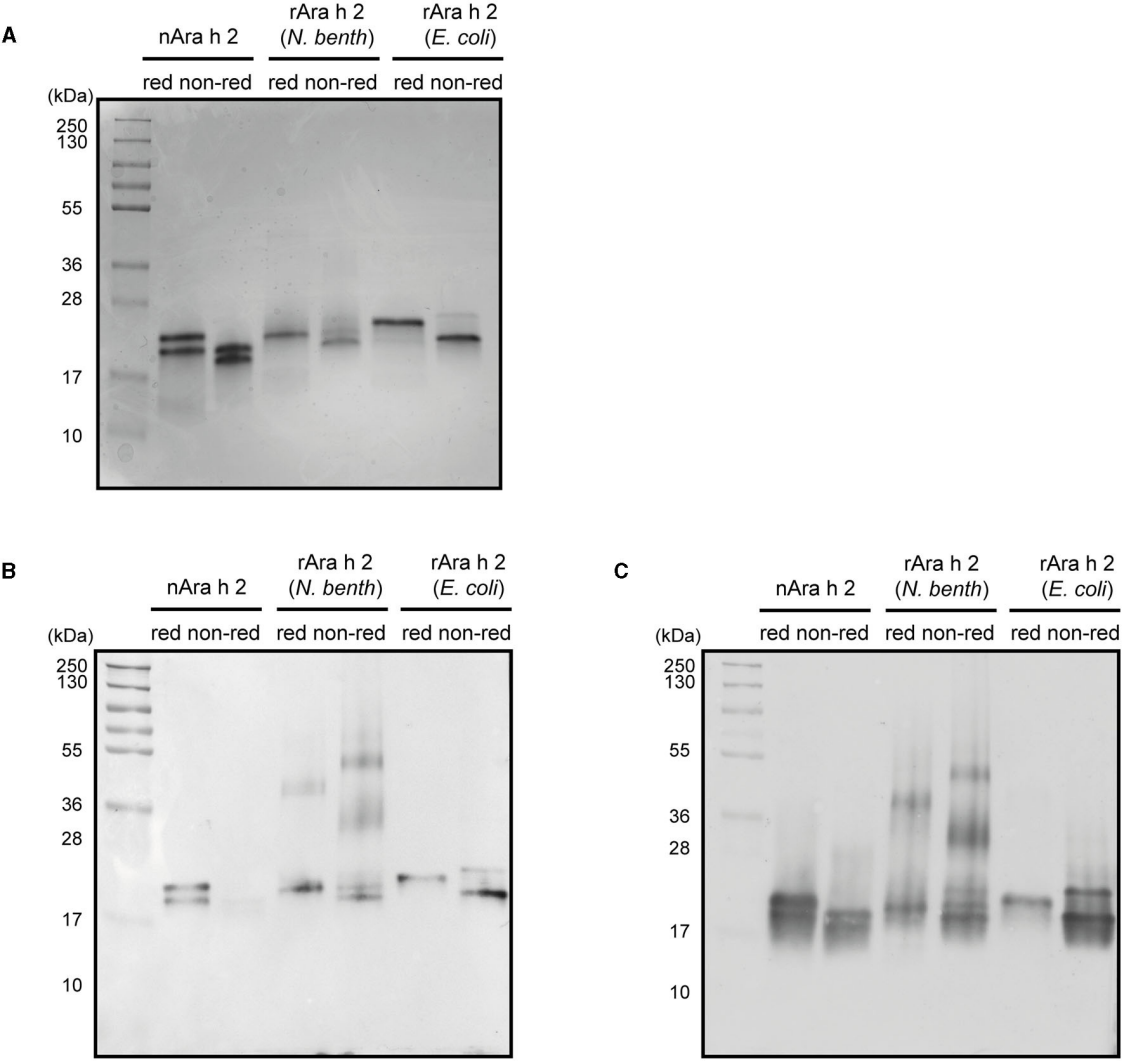
Characterization of nAra h 2, rAra h 2 (*N. benthamiana*), and rAra h 2 (*E. coli)*. **(A)** The purity of nAra h 2, rAra h 2 (*N. benthamiana*), and rAra h 2 (*E. coli*) was visualized by CBB-stain after running 2 μg protein per lane on SDS-PAGE under reducing (red) and non-reducing (non-red) conditions. **(B)** All three proteins were detected using a monoclonal anti-Ara h 2 antibody, loaded as in **(A)**. **(C)** A pool of sera of peanut-allergic patients (*n* = 5) was used to detect all Ara h 2 proteins, loaded as in **(A)**.

All three proteins had similar alpha-helical secondary structures as measured by circular dichroism spectroscopy (Figure 2A). Dynamic light scattering analysis revealed monomeric states for all three proteins (Figure 2B). The rAra h 2 from *E. coli* contained 1% aggregates. To assess their purity, all proteins were run under reducing and non-reducing conditions on SDS-PAGE (Figure 3A). All non-reduced proteins migrated at their correct sizes on SDS-PAGE (Table 1, Figure 3A). Detection of all proteins by an anti-Ara h 2 monoclonal antibody revealed additional faint bands at sizes corresponding to dimeric and trimeric forms of *N. benthamiana*-expressed rAra h 2 (Figure 3B). nAra h 2 could not be detected by this monoclonal antibody when separated under non-reducing conditions, possibly due to the altered net charge of the non-reduced protein. When a pool of sera from peanut-allergic patients was used, all proteins were detected strongly by visualizing bound IgE under both reducing and non-reducing conditions as a result of the polyclonal antibody responses of the patients (Figure 3C).

### rAra h 2 Expressed in *N. benthamiana* Leaves Harbors Hydroxyprolines

Mass spectrometry analysis of rAra h 2 (*N. benthamiana*) was performed to identify contaminants and to detect PTMs. rAra h 2 was detected with 89.6% sequence coverage and with the highest score among all detected proteins, indicating high confidence of the match (Table 2). Besides, contaminants from *Homo sapiens* due to sample handling and from *N. benthamiana* were detected with lower score values in comparison to the one of Ara h 2. *N. benthamiana*-made rAra h 2 harbored more hydroxyprolines (P23, P43. P46, P50, P53, P65, and P67) than the nAra h 2 (P46, P53, and P65). Six out of seven hydroxyproline residues passed the manual confirmation (Table 3). Posterior error probability (PEP) values indicated low possibilities of a false positive discovery for all hydroxyprolines. The ratio of modified peptide over baseline (mod/base) indicated a mix of modified and non-modified peptides. Accordingly, only two proline residues were hydroxylated substantially (P46, P53), since minimum 50% of the found peptides carried this modification (Table 3). Interestingly, there were three additional hydroxyprolines present in the plant-made recombinant (P43, P50, and P67) in comparison to nAra h 2 (Table 4). The search for HexNAc attachments on serine/threonine residues did not reveal any modified sites, indicating the absence of potential O-glycosylation of the rAra h 2 from *N. benthamiana* (PRIDE repository dataset identifier: PXD027015).

**Table 2:** Relevant proteins found in the rAra h 2 sample expressed in *N. benthamiana* leaves, as detected by mass spectrometry.

**Protein names**	**Origin**	**Sequence coverage (%)**	**Score**
Ara h 2	*Arachis hypogaea*	89.6	323.3
Keratin	*Homo sapiens*	42.6	323.3
Hornerin	*Homo sapiens*	7.3	69.2
Filaggrin	*Homo sapiens*	5.4	61.2
Actin	*N. benthamiana*	8.4	13.0
Histone H4	*N. benthamiana*	21.4	12.1
Tubulin alpha-2 chain	*N. benthamiana*	4.7	9.3

*Protein scores are calculated by summing the individual scores of corresponding peptides*.

**Table 3:** Hydroxyproline residues found in *N. benthamiana*-made rAra h 2.

**Hydroxyproline positions**	**Manual validation**	**Localization probability**	**Posterior error possibility (PEP)**	**Score**	**Ratio (mod/base) (%)**
P23	Did not pass	1.0	0.000175	104.2	2.7
P43	Passed	1.0	0.000144	84.9	3.0
P46	Passed	1.0	4.29E-46	324.8	127.2
P50	Passed	1.0	0.000144	84.9	1.1
P53	Passed	1.0	4.29E-46	324.8	63.1
P65	Passed	1.0	2.82E-16	218.1	6.5
P67	Passed	1.0	1.95E-15	208.0	0.3

*Hydroxyproline residues are numbered excluding the N-terminal methionine. Localization probability indicates the particular site of hydroxylation and is calculated from the MS/MS spectrum. A localization probability >0.75 means a highly confident site. PEP value indicates the probability of incorrect matches in the peptide spectrum. Smaller PEP values mean lower chances of a false ID. Mod/base ratios are calculated from the ion intensities of modified peptides divided by the intensities of non-modified peptides*.

**Table 4:** Comparison of manually confirmed site-specific hydroxylations of proline residues in rAra h 2 from *N. benthamiana* and in the natural allergen.

**Hydroxyproline positions**	**Adjacent sequence**	**In other plant species**	***Arachis hypogaea***	***Nicotiana benthamiana***
43	DPY	n.k.	No	Yes
46	SPS	Yes	Yes	Yes
50	DPY	n.k.	No	Yes
53	SPS	Yes	Yes	Yes
65	SPS	Yes	Yes	Yes
67	SPY	n.k.	No	Yes

*Hydroxyproline residues of N. benthamiana-produced rAra h 2 are numbered excluding the N-terminal methionine*.

### rAra h 2 Produced in *N. benthamiana* Is a Viable Alternative to the *E. coli*-Made Product in ELISA

**Figure 4:**
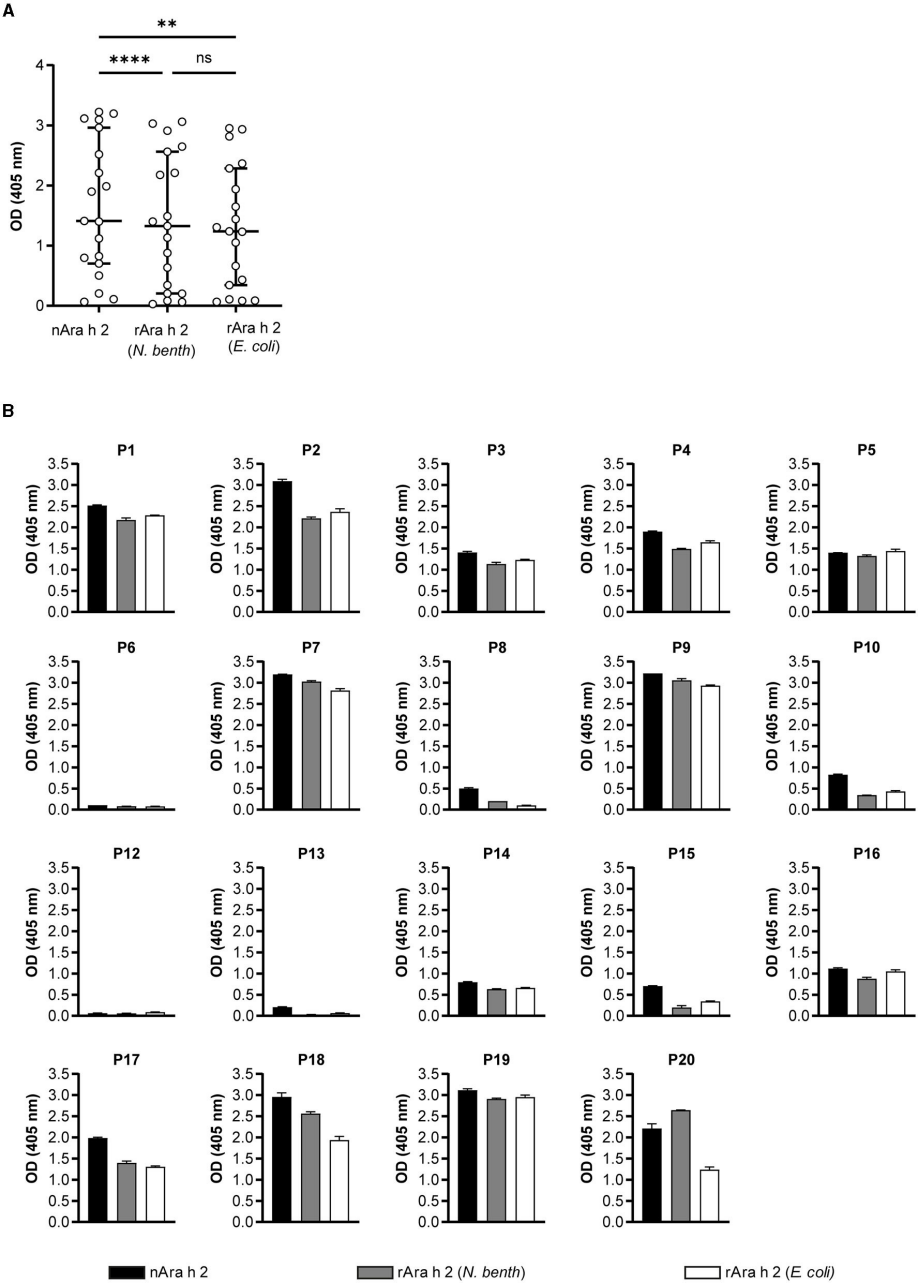
IgE-binding of Ara h 2 proteins compared by ELISA. **(A)** IgE-binding to rAra h 2 from *N. benthamiana* was measured and compared with the nAra h 2 and the rAra h 2 from *E. coli*, using sera from 19 peanut-allergic patients. Data are the means of technical duplicates (*n* = 2). ^****^P ≤ 0.0001, ^**^P ≤ 0.01, ns: *P* > 0.05. **(B)** IgE-binding to all three Ara h 2 proteins was determined for each patient. Error bars represent the standard deviation from technical duplicates (*n* = 2).

The IgE-binding capacities of rAra h 2 from *N. benthamiana* and *E. coli*, as well as the natural allergen, were investigated by IgE ELISA (Figure 4). In a cohort of 20 peanut-allergic patients (demonstrated by case history, skin prick test, and ImmunoCAP; see Supplementary Table 1), IgE of 19 patients recognized nAra h 2 in ELISA. For Patient 11 (Ara h 2 ImmunoCAP = 1.1 kU/L), the results were below the detection limit; hence, data obtained from this patient were excluded from Figure 4. nAra h 2 showed the highest IgE-binding capacity (median OD_405_ = 1.41), which was significantly higher than both *N. benthamiana*-produced (median OD_405_ = 1.33, *P* < 0.0001) and *E. coli*-produced allergens (median OD_405_ = 1.24, *P* = 0.002; Figure 4A). Interestingly, the median-bound IgE value of the plant-made rAra h 2 did not differ significantly from the recombinant from *E. coli* (*P* = 0.99).

Patient-specific IgE recognition of all Ara h 2 proteins is shown in Figure 4B. Four out of 19 patients (P6, P8, P12, and P13) had very low mean OD_405_ values for the recombinant Ara h 2 proteins. Notably, patients P7, P9, P17, P18, and P20 had mean OD_405_ values for IgE-binding to plant-made rAra h 2 that were higher than the values obtained for the *E. coli* recombinant protein (Figure 4B). In contrast, rAra h 2 from *E. coli* demonstrated stronger IgE-binding than the one from *N. benthamiana* for eight patients (P1, P2, P3, P4, P5, P10, P15, and P16). Additionally, we investigated the correlation between ELISA results for all three proteins and sIgE values of patients measured to Ara h 2 by ImmunoCAP (Supplementary Figure 2). Significantly positive correlations (measured by Spearman's r) were observed for each protein (*r* = 0.92–0.95, *P* < 0.0001).

The ratios of IgE-binding values between all three pairs of proteins were calculated and plotted against ImmunoCAP values representing the sIgE concentrations of the patients (Supplementary Figure 3). The ratio of IgE-binding to rAra h 2 (*N. benthamiana*) and to nAra h 2 correlated significantly with the ImmunoCAP values (Supplementary Figure 3A, *r* = 0.55, *P* = 0.0139). On the other hand, there was no significant correlation between sIgE concentrations of patients and the ratio of IgE-binding to rAra h 2 (*E. coli*) and to nAra h 2 (Supplementary Figure 3B, *r* = 0.39, *P* = 0.0975). IgE from patients with lower sIgE concentrations (ImmunoCAP <50 kU_A_/L) bound to nAra h 2 better than to either of the recombinants (Supplementary Figures 3A,B). Although there was no significant correlation with ImmunoCAP values (*r* = 0.42, *P* = 0.0765), the ratio of IgE-binding to rAra h 2 (*N. benthamiana*) and to rAra h 2 (*E. coli*) was >1 for three patients, who had lower sIgE levels (P8, P18, and P20, Supplementary Figure 3C).

### Hydroxyproline Residues Are Involved in Efficient Cross-Linking of IgE

**Figure 5:**
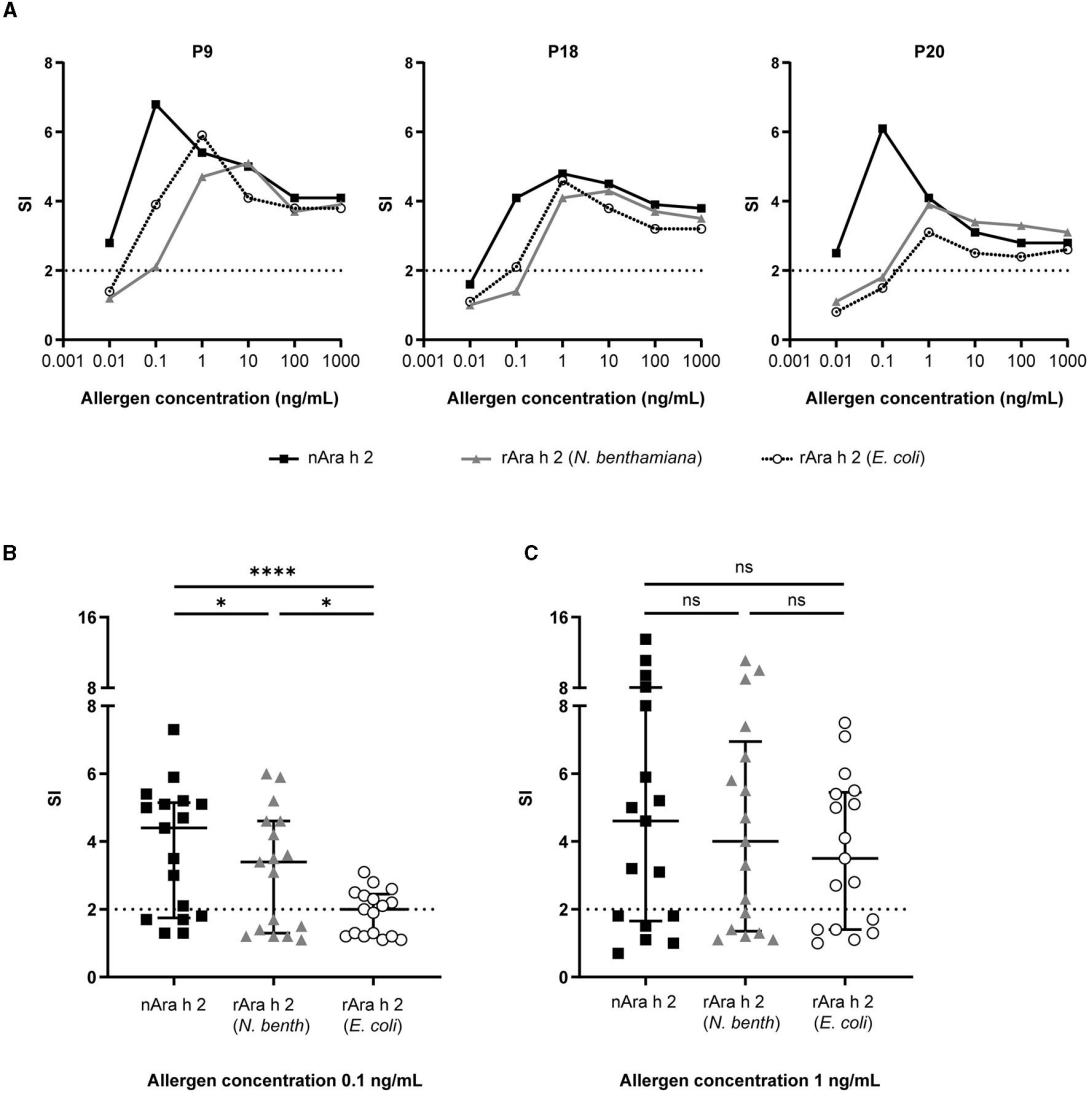
RBL assays were performed to investigate the IgE cross-linking capacity of rAra h 2 from *N. benthamiana* in comparison with the nAra h 2 and the rAra h 2 from *E. coli*. **(A)** Three patients (P9, P18, and P20) were selected to perform the allergen titrations. Data are the means of technical triplicates (*n* = 3). Data are represented as stimulation indices (SI) calculated by dividing the luminescence values from antigen-treated cells with the ones from non-stimulated cells. A value above 2 was considered positive (indicated by dotted lines). **(B)** Data obtained from 17 peanut-allergic patients were used to compare the IgE-cross-linking performances of all Ara h 2 proteins at 0.1 ng/mL allergen concentration. Median values and interquartile range are indicated. ^****^P ≤ 0.0001, ^*^P ≤ 0.05. **(C)** As in **(B)** at 1 ng/mL concentration. ns: *P* > 0.05.

The IgE-cross-linking capacities of both rAra h 2 products were compared with nAra h 2 by BAT using humanized RBL cells (Figure 5). The results are presented as SI in comparison to the activation of untreated cells. To assess the optimal concentration for all allergens, RBL cells were passively sensitized with IgE from sera of three allergic patients (P9, P18, and P20) and stimulated with all proteins separately in concentrations from 0.01 to 1,000 ng/mL (Figure 5A). The two most efficient concentrations, namely 0.1 and 1 ng/mL, were chosen for testing all 20 sera.

Three serum samples (P3, P4, and P7) of 20 failed to efficiently sensitize the cells (shown by an SI <2 upon stimulation with anti-IgE as positive control). Data obtained from these patients are not included in Figure 5. nAra h 2 did not activate basophils sensitized with sera from five patients (Supplementary Figure 4A), while both recombinant allergens were not capable of activating basophils sensitized with sera from six patients (Supplementary Figures 4B,C). When stimulated with 0.1 ng/mL of allergen, basophil activation was significantly more efficient with nAra h 2 (median SI = 4.4) than with the rAra h 2 from *N. benthamiana* (median SI = 3.4, *P* = 0.018) or the rAra h 2 from *E. coli* (median SI = 2.0, *P* < 0.0001; Figure 5B). On the other hand, rAra h 2 from *N. benthamiana* crosslinked receptor-bound IgE (median SI = 3.4) almost twice as much as the one from *E. coli* (median SI = 2.0, *P* = 0.039; Figure 5B). The stimulation index almost doubled for most patients when the allergen concentration was increased from 0.1 to 1 ng/mL for all Ara h 2 proteins (Supplementary Figure 4). At 1 ng/mL, the IgE cross-linking capacities of neither rAra h 2 (*N. benthamiana*) (median SI = 4.0) nor rAra h 2 (*E. coli*) (median SI = 3.5) were significantly different from the nAra h 2 (median SI = 4.6) (Figure 5C). Interestingly, the median SI induced by 1 ng/mL of rAra h 2 from *E. coli* was close to the one induced by 0.1 ng/mL of *N. benthamiana*-produced allergen, indicating the potency of plant-made rAra h 2 to activate the cells at a lower concentration (Figure 5C).

To investigate these observations together with sIgE values of patients, we plotted ratios of SI for each allergen (at 0.1 ng/mL) against the ImmunoCAP values (Supplementary Figure 5). There was no significant correlation between the ratio of the SIs of rAra h 2 (*N. benthamiana*) and nAra h 2 and the sIgE values of the patients (Supplementary Figure 5A, *r* = 0.04, *P* = 0.87). However, the ratio of the SIs of rAra h 2 (*E. coli*) and nAra h 2 decreased significantly when the sIgE levels of patients increased (Supplementary Figure 5B, *r* = – 0.80, *P* = 0.0002). In contrast, a strong positive correlation was observed when the ratio of SI for rAra h 2 (*N. benthamiana*) and for rAra h 2 (*E. coli*) was plotted against the sIgE levels of patients (Supplementary Figure 5C, *r* = 0.85, *P* < 0.0001).

## Discussion

We produced the major peanut allergen Ara h 2, a 2S albumin seed storage protein, in the ER of *N. benthamiana* leaf cells by employing a plant virus-based transient expression system and studied its biochemical and immunological characteristics. *Agrobacterium tumefaciens*-mediated expression in *N. benthamiana* plants yielded 200 mg rAra h 2 per kg biomass within 10 days. Site-specific proline hydroxylation was observed within the DPYSPS repeats of rAra h 2 expressed in *N. benthamiana*. Unlike the natural protein, which has three hydroxyprolines at the SPS sequences, *N. benthamiana*-made rAra h 2 was modified at six proline residues within the SPS, DPY, and SPY sequences. The involvement of these PTMs in IgE-binding and cross-linking was investigated using immunological assays. For this purpose, *N. benthamiana*-produced Ara h 2 was compared with the hydroxyproline-free recombinant expressed in *E. coli* and with the natural allergen isolated from peanuts. Natural Ara h 2 outcompeted both of the recombinant proteins in all immunological assays used. Although neither of the recombinant proteins outperformed the other in ELISA, IgE-cross-linking results obtained from BAT revealed a remarkable superiority of the *N. benthamiana*-produced allergen over the *E. coli* product. Thus, a higher similarity of the plant-made rAra h 2 to the natural protein was shown. The *E. coli-*produced protein was co-expressed with the disulfide-bond isomerase DsbC, therefore, harbored disulfide bridges (Lobstein et al., 2012) but no hydroxyprolines. Thus, it is highly likely that the observed differences in IgE-binding and cross-linking ability between the two recombinant proteins resulted from this modification.

We expressed rAra h 2 using the magnICON^®^ system (Supplementary Figure 1), which enables easier handling during infection of the leaves by omitting *in vitro* transcription steps as employed previously (Krebitz et al., 2000). The highest yield ever achieved for transient protein expression in *N. benthamiana* was reported for the cytosolic production of GFP at 5 g/kg fresh biomass (Marillonnet et al., 2004). In the case of allergen production, deconstructed geminiviral vectors were utilized for cytosolic transient expression of rBet v 1 at 1.2 g/kg fresh biomass (Yamada et al., 2020). This is six-fold higher than our yield of the rAra h 2 expression in the ER (Table 1). Expression yields in *N. benthamiana* leaves vary greatly, depending on the protein of interest as well as subcellular targeting. The highest yield achieved for the porcine circovirus 2 capsid protein was obtained for the expression in chloroplasts (102 mg/kg fresh biomass), followed by expression in the cytosol (6.5 mg/kg) and the ER (0.65 mg/kg) (Park et al., 2021). Furthermore, endogenous proteases of *N. benthamiana*, which may be located in the cytosol or post-ER compartments (Castilho et al., 2014), have a negative impact on recombinant protein production in plant cells, which can be dampened by depleting proteases *via* the CRISPR/Cas9-mediated knockout (Jutras et al., 2020). The insulin analog SCI-57 was transiently expressed in the ER with a yield reaching up to 150 mg/kg fresh biomass (Munoz-Talavera et al., 2019), which is in the range of our yield obtained with rAra h 2 production in the ER (200 mg/kg). Finally, efficient cell lysis is decisive for extracting all the expressed protein into the soluble fraction, as 60% of the rAra h 2 remained in the insoluble fraction which affected our yield (Supplementary Figure 1D). Our results represent a lab-scale production. By utilizing large-scale manufacturing techniques, infiltration of magnICON^®^ vectors into kilograms of plants per hour is feasible (Klimyuk et al., 2014). Based on the capacity of a commercial-scale manufacturing facility with infiltration of 180–360 tons of biomass per year, 35–70 kg of rAra h 2 could be produced (Holtz et al., 2015). According to calculations done with SuperPro Designer modeling software, which were based on the manufacturing cost of butyrylcholinesterase produced in *N. benthamiana*, one gram of rAra h 2 transiently produced with a yield of 200 mg/kg would cost around US$ 1,000 (Tusé et al., 2014). By optimizing the yield to 500 mg/kg, costs could be reduced to ~US$ 600 per gram of protein (Tusé et al., 2014).

N-glycosylation is initiated in the ER membrane, whereas O-glycosylation mainly occurs later in the Golgi apparatus (Strasser, 2016). Interestingly, when expressed in the cytosol, a partial glycosylation was observed for rBet v 1, which harbors a canonical N-glycosylation site (Yamada et al., 2020). By terminating the secretion in the ER and mutating the canonical site, we could avoid any O- or N-linked glycosylation of the rAra h 2, respectively (PRIDE repository dataset identifier: PXD027015).

We report the hydroxylation of the proline residue in the repeating SPS sequences for rAra h 2 expressed in *N. benthamiana* leaves. The natural allergen harbors only three hydroxyproline residues, which are all located in the SPS motifs (Figure 1A and Table 1; Li et al., 2010). The recognition sequences for site-specific hydroxylation of proline residues have not been completely identified. Yet, a review summarized the observed sequence motifs for proline hydroxylation in several plant species (Canut et al., 2016). Membrane-anchored prolyl-4-hydroxylases (P4H) are responsible for modifying proline residues into *trans*-4-L-proline in *N. tabacum* (Yuasa et al., 2005), which is likely similar to the mechanism in *N. benthamiana*. Recently, four paralogs of P4H of *N. benthamiana* have been studied for their substrate specificity and subcellular localization (Mócsai et al., 2021). All paralogs were found to localize to the Golgi apparatus with the exception of P4H4, which was also found in the ER. Most probably, this paralog was responsible for the proline hydroxylation in our case, as we terminated the rAra h 2 expression in the ER. For *Nicotiana alata* and *N. tabacum*, the SPS sequence was reported to be favored for proline hydroxylation (Du et al., 1994; Youl et al., 1998; Shpak et al., 1999). Although detected with low-modification ratios, the additional hydroxyproline residues at the DPY and SPY sequences (P50 and P67, respectively) observed for *N. benthamiana*-produced rAra h 2 have not been reported for any plant species before (Table 4). Importantly, only two prolines out of six were hydroxylated substantially, as at least 50% of all found peptides were modified at these locations (Table 3). Our results contribute to the knowledge of site-specific proline hydroxylation motifs and to their modification frequencies observed in *N. benthamiana*. As hydroxylation of proline residues can be subjected to O-glycosylation for certain proteins (Petersen et al., 2021), understanding site-specific modifications in various species plays a crucial role when expressing recombinant proteins with pharmaceutical value. PTMs, such as hydroxylation, glycosylation, and methylation, are often used for enhancing the bioactivity, stability, and immunogenicity of the products (Mathieu-Rivet et al., 2020). For drug designs, it is of great interest to decipher site-specific O-glycosylation since their presence on peptide hormones regulates vital biological activities (Madsen et al., 2020).

Producing properly folded rAra h 2 is important, as conformational epitopes are major contributors to its binding of IgE from sera of peanut-allergic patients (Tscheppe et al., 2020). So far, there have been only two reports of properly folded rAra h 2; (i) in the *Trichoplusia ni* BTI-TN5B1-4 “high five” insect cells (Tscheppe et al., 2020), (ii) in the Origami (DE3) *E. coli* strain (Lehmann et al., 2003). We report correctly folded rAra h 2 protein from *N. benthamiana* plants and from SHuffle^®^ T7 Express lysY *E. coli* strain (Figure 2A). After confirming natural-like structures for both recombinant proteins, we investigated their reactivity to monoclonal and polyclonal antibodies in comparison to the natural allergen (Figures 3B,C, 4). When allergens are coated onto a surface for direct binding by polyclonal IgE, a single epitope suffices for generating a positive signal. Hence, we did not observe major differences between the two recombinants from *N. benthamiana* and *E. coli* in ELISA (Figure 4A). In line with the data published so far (Gregory et al., 2016; Tscheppe et al., 2020), the natural allergen outcompeted the recombinant proteins expressed in *N. benthamiana* and *E. coli* (Figure 4A).

Interestingly, only four (P5, P14, P16, and P19) out of 19 patients (21%) reacted to the recombinantly produced Ara h 2 proteins almost as well as the natural protein (Figure 4B). The IgE-binding was especially low for the natural and recombinant Ara h 2 proteins for patients P6, P8, P12, and P13, possibly due to their low sIgE levels (Figure 4B, Supplementary Table 1). Based on the ratios of IgE-binding to *N. benthamiana*-made rAra h 2 and to *E. coli*-made rAra h 2, we concluded that patients with lower sIgE values favored the plant-made recombinant allergen over the *E. coli* product (Supplementary Figure 3C). Lower-serum levels of sIgE may result in lesser polyclonality, highlighting the importance of each specific epitope for that patient. We hypothesize that hydroxyprolines at P43, P46, P50, P53, and P65, which are located within the immunodominant IgE epitope DPYSPS (Figure 1B), are more crucial for IgE-binding of patients with lower sIgE levels. A recent study has compared IgE-binding to 33 overlapping peptides of Ara h 2 in peanut-allergic and peanut-sensitized tolerant groups (Santos et al., 2020). Interestingly, trends of IgE-binding to all peptides by two patient groups were similar for all sIgE levels, yet IgG4/IgE ratios to the peptides-harboring DPYSPS motif were lower for peanut-allergic patients in comparison to the sensitized but tolerant group. When testing the *N. benthamiana*-produced rAra h 2, sera of peanut-tolerant patients could provide further information on the role of hydroxyprolines in IgE and IgG4 responses.

Next, we used the well-established RBL cell line RS-ATL8 (Nakamura et al., 2010; Kalic et al., 2020) to measure the IgE-cross-linking capacity of the Ara h 2 proteins. In this assay, strong cell activation depends on the number of accessible epitopes, their proximity and the affinity of IgE to these epitopes. The most immunogenic linear epitope combination for Ara h 2 has been reported in a liposomal nanoallergen display (Deak et al., 2017). The authors showed the importance of the hydroxyproline-containing linear epitope for degranulating RBL cells in combination with the epitopes at residues 1–6 and 108–116. Ara h 2 is a highly potent allergen able to stimulate basophils at ng/mL concentrations (Palmer et al., 2005; Anzengruber et al., 2017; Chapuis et al., 2018). We tested concentrations from 0.01 to 1,000 ng/mL (Figure 5A). The optimal allergen concentrations were 0.1 and 1 ng/mL. Although to a significantly lesser extent than nAra h 2, rAra h 2 from *N. benthamiana* activated basophils more efficiently than the *E. coli* recombinant already at the lowest concentration (Figure 5B, Supplementary Figures 5B,C), and this trend continued at 1 ng/mL (Figure 5C). The hydroxyproline-containing rAra h 2 may have a higher affinity for IgE, which could increase the lifetime of IgE-allergen complexes, hence enhancing the basophil activation by *N. benthamiana*-made rAra h 2.

Notably, nAra h 2 outcompeted both recombinant proteins in all immunological assays used (Figures 4A, 5B,C), which may be due to the presence of several isoforms and the combination of all structural variations among them. Unless nAra h 2.0201 is isolated individually from the naturally occurring isoform mixture, it is not possible to objectively compare the recombinant allergens with their natural counterpart. Moreover, heat processing of peanut results in advanced glycation end products *via* Maillard reactions, and those modifications are major contributors to the IgE reactivity of Ara h 2 (Vissers et al., 2011). These thermal processing reactions were even shown to enhance the IgE reactivity of *E. coli*-produced rAra h 2 (Gruber et al., 2005). Thermal processing of the *N. benthamiana*-made rAra h 2 shall be investigated in comparison to the natural allergen as a future perspective of this study. In addition, both recombinant allergens carried hexa-histidine tags for easy purification (Figures 1B,C). A single amino acid mutation at N106Q and a C-terminal ER-retention signal was present in the *N. benthamiana*-made Ara h 2 (Figure 1B), whereas an N-terminal eight residue-long extension was present in the *E. coli*-made protein (Figure 1C). Such alterations and mutations in the amino acid sequence might interfere with the natural fold of proteins or with their antibody-binding capacity, which has to be considered when comparing rAra h 2 proteins to the natural allergen.

To date, both natural and rAra h 2 have been used in routine singleplex and multiplex diagnostic tests to detect sIgE in sera from patients, which is regarded as an indicator of a high risk for developing strong allergic symptoms to peanut (Breiteneder et al., 2020). As peanut allergy is a life-threatening disease, accurate diagnosis is vital. Recombinant allergens and mixtures thereof have become more preferable than natural extracts due to the ease of standardization (Valenta et al., 2018b). The quality of BAT, still under development for routine testing, relies on the presence of all naturally occurring epitopes (Krogulska and Wood, 2020). Our study has shown that the peanut major allergen Ara h 2 expressed in *N. benthamiana* plants displayed an equal or superior performance in immunological assays in comparison to Ara h 2 produced in a prokaryotic system. Future perspectives include testing the *N. benthamiana*-made rAra h 2 with peanut-sensitized but tolerant patients in comparison to a peanut-allergic patient cohort and investigating the involvement of PTMs in allergen-binding affinities to IgG4 and IgE (Santos et al., 2020). Finally, our data present future perspectives for eukaryotic expression of hypoallergenic Ara h 2 variants for use in allergen-specific immunotherapy.

## Data Availability Statement

The mass spectrometry proteomics data have been deposited to the ProteomeXchange Consortium via PRIDE partner repository (Perez-Riverol et al., 2019) with the dataset identifier PXD027015 (https://www.ebi.ac.uk/pride/archive/projects/PXD027015).

## Ethics Statement

The studies involving human participants were reviewed and approved by Ethics Committee of Lower Austria (GS4-EK-4/503-2017) and conducted in accordance with the Declaration of Helsinki. The patients/participants provided their written informed consent to participate in this study.

## Author Contributions

ÖÜ and TK designed and performed experiments. ÖÜ, TK, CR, and HB analyzed the data. VM and NL assisted in the experiments. AT produced reagents and performed experiments. CH and WH provided sera from patients. HB provided input and supervised experiments. ÖÜ and HB interpreted the data and wrote the manuscript. All the authors read the manuscript.

## Funding

This study was supported by the Austrian Science Fund (FWF) grants MCCA W1248-B30 and P 30936-B30 and the Danube Allergy Research Cluster project P06 funded by the Country of Lower Austria.

## Conflict of Interest

The authors declare that the research was conducted in the absence of any commercial or financial relationships that could be construed as a potential conflict of interest.

## Publisher's Note

All claims expressed in this article are solely those of the authors and do not necessarily represent those of their affiliated organizations, or those of the publisher, the editors and the reviewers. Any product that may be evaluated in this article, or claim that may be made by its manufacturer, is not guaranteed or endorsed by the publisher.

## Abbreviations

AP: alkaline phosphatase
BAT: basophil activation test
BCA: bicinchoninic acid assay
CBB: Coomassie Brilliant Blue R-250
ELISA: enzyme-linked immunosorbent assay
ER: endoplasmic reticulum
IgE: immunoglobulin E
LB: left T-DNA border
MP: movement protein
OD: optical density
PEP: posterior error probability
PTM: posttranslational modifications
RBL: rat basophil leukemia
RB: right T-DNA border
RdRP: RNA-dependent RNA polymerase
SI: stimulation index
sIgE: allergen-specific IgE
SP: signal peptide
T-DNA: transfer DNA
TMV: tobacco mosaic virus.

## References

[B1] Anzengruber J. Bublin M. Bonisch E. Janesch B. Tscheppe A. Braun M. L.. (2017). Lactobacillus buchneri S-layer as carrier for an Ara h 2-derived peptide for peanut allergen-specific immunotherapy. Mol. Immunol. 85, 81–88. 10.1016/j.molimm.2017.02.005 28212503 PMC5386144

[B2] Apostolovic D. Marsh J. T. Baumert J. Taylor S. L. Westphal A. De Jongh H.. (2021). Purification and initial characterization of Ara h 7, a peanut allergen from the 2S albumin protein family. J. Agric. Food Chem. 69, 6318–6329. 10.1021/acs.jafc.1c00618 34037388

[B3] Astier C. Morisset M. Roitel O. Codreanu F. Jacquenet S. Franck P.. (2006). Predictive value of skin prick tests using recombinant allergens for diagnosis of peanut allergy. J. Allergy Clin. Immunol. 118, 250–256. 10.1016/j.jaci.2006.04.053 16815163

[B4] Barral P. Batanero E. Villalba M. Rodriguez R. (2005). Expression of the major olive pollen allergen Ole e 10 in the yeast *Pichia pastoris*: evidence of post-translational modifications. Protein Expr. Purif. 44, 147–154. 10.1016/j.pep.2005.04.012 15935694

[B5] Bernard H. Guillon B. Drumare M. F. Paty E. Dreskin S. C. Wal J. M.. (2015). Allergenicity of peanut component Ara h 2: Contribution of conformational versus linear hydroxyproline-containing epitopes. J. Allergy Clin. Immunol. 135, 1267–1274. 10.1016/j.jaci.2014.10.025 25483599

[B6] Bernard H. Meisel H. Creminon C. Wal J. M. (2000). Post-translational phosphorylation affects the IgE binding capacity of caseins. FEBS Lett. 467, 239–244. 10.1016/S0014-5793(00)01164-9 10675546

[B7] Breiteneder H. Krebitz M. Wiedermann U. Wagner B. Essl D. Steinkellner H.. (2001). Rapid production of recombinant allergens in *Nicotiana benthamiana* and their impact an diagnosis and therapy. Int. Arch. Allergy Immunol. 124, 48–50. 10.1159/000053665 11306923

[B8] Breiteneder H. Peng Y. Q. Agache I. Diamant Z. Eiwegger T. Fokkens W. J.. (2020). Biomarkers for diagnosis and prediction of therapy responses in allergic diseases and asthma. Allergy 75, 3039–3068. 10.1111/all.14582 32893900 PMC7756301

[B9] Bublin M. Radauer C. Wilson I. B. Kraft D. Scheiner O. Breiteneder H.. (2003). Cross-reactive N-glycans of Api g 5, a high molecular weight glycoprotein allergen from celery, are required for immunoglobulin E binding and activation of effector cells from allergic patients. FASEB J. 17, 1697–1699. 10.1096/fj.02-0872fje 12958180

[B10] Canut H. Albenne C. Jamet E. (2016). Post-translational modifications of plant cell wall proteins and peptides: a survey from a proteomics point of view. Biochim. Biophys. Acta 1864, 983–990. 10.1016/j.bbapap.2016.02.022 26945515

[B11] Castilho A. Windwarder M. Gattinger P. Mach L. Strasser R. Altmann F.. (2014). Proteolytic and N-glycan processing of human alpha1-antitrypsin expressed in *Nicotiana benthamiana*. Plant Physiol. 166, 1839–1851. 10.1104/pp.114.250720 25355867 PMC4256845

[B12] Chan C. J. Yong Y. S. Song A. A. L. Abdul Rahim R. In L. L. Lim R. L. H. (2020). *Lactococcus lactis* harbouring Ara h 2.02 alleviates allergen-specific Th2-associated responses in sensitized mice. J. Appl. Microbiol. 128, 862–874. 10.1111/jam.14524 31758869

[B13] Chapuis A. Thevenot J. Coutant F. Messaoudi K. Michaud E. Pereira B.. (2018). Ara h 2 basophil activation test does not predict clinical reactivity to peanut. J. Allergy Clin. Immunol. Pract. 6, 1772–1774. 10.1016/j.jaip.2018.01.021 29410305

[B14] Chen M. H. Huang L. F. Li H. M. Chen Y. R. Yu S. M. (2004). Signal peptide-dependent targeting of a rice alpha-amylase and cargo proteins to plastids and extracellular compartments of plant cells. Plant Physiol. 135, 1367–1377. 10.1104/pp.104.042184 15235120 PMC519054

[B15] Chinthrajah R. S. Cao S. Dunham T. Sampath V. Chandra S. Chen M.. (2020). Oral immunotherapy for peanut allergy: the pro argument. World Allergy Organ. J. 13:100455. 10.1016/j.waojou.2020.100455 33005286 PMC7519204

[B16] Cox J. Mann M. (2008). MaxQuant enables high peptide identification rates, individualized p.p.b.-range mass accuracies and proteome-wide protein quantification. Nat. Biotechnol. 26, 1367–1372. 10.1038/nbt.1511 19029910

[B17] Curin M. Garib V. Valenta R. (2017). Single recombinant and purified major allergens and peptides How they are made and how they change allergy diagnosis and treatment. Ann. Allergy Asthma Immunol. 119, 201–209. 10.1016/j.anai.2016.11.022 28890016 PMC6390930

[B18] Daniell H. Kulis M. Herzog R. W. (2019). Plant cell-made protein antigens for induction of oral tolerance. Biotechnol. Adv. 37:107413. 10.1016/j.biotechadv.2019.06.012 31251968 PMC6842683

[B19] Deak P. E. Vrabel M. R. Kiziltepe T. Bilgicer B. (2017). Determination of crucial immunogenic epitopes in major peanut allergy protein, Ara h2, via novel nanoallergen platform. Sci. Rep. 7:3981. 10.1038/s41598-017-04268-6 28638052 PMC5479826

[B20] Du H. Simpson R. J. Moritz R. L. Clarke A. E. Bacic A. (1994). Isolation of the protein backbone of an arabinogalactan-protein from the styles of *Nicotiana alata* and characterization of a corresponding cDNA. Plant Cell 6, 1643–1653. 10.1105/tpc.6.11.1643 7827496 PMC160550

[B21] Engler C. Kandzia R. Marillonnet S. (2008). A one pot, one step, precision cloning method with high throughput capability. PLoS ONE 3:e3657. 10.1371/journal.pone.0003647 18985154 PMC2574415

[B22] Frohner I. E. Mudrak I. Schuchner S. Anrather D. Hartl M. Sontag J. M.. (2020). PP2AC Phospho-Tyr(307) antibodies are not specific for this modification but are sensitive to other PP2AC modifications including Leu(309) methylation. Cell Rep. 30, 3171–3182. 10.1016/j.celrep.2020.02.035 32130916

[B23] Gadisseur R. Chapelle J. P. Cavalier E. (2011). A new tool in the field of *in-vitro diagnosis* of allergy: preliminary results in the comparison of ImmunoCAP(c) 250 with the ImmunoCAP(c) ISAC. Clin. Chem. Lab. Med. 49, 277–280. 10.1515/CCLM.2011.052 21143018

[B24] Giritch A. Klimyuk V. Gleba Y. (2017). 125 years of virology and ascent of biotechnologies based on viral expression. Cytol. Genet. 51, 87–102. 10.3103/S0095452717020037 30484616

[B25] Gleba Y. Y. Tuse D. Giritch A. (2014). Plant viral vectors for delivery by Agrobacterium. Curr. Top. Microbiol. Immunol. 375, 155–192. 10.1007/82_2013_352 23949286

[B26] Gregory J. A. Shepley-Mctaggart A. Umpierrez M. Hurlburt B. K. Maleki S. J. Sampson H. A.. (2016). Immunotherapy using algal-produced Ara h 1 core domain suppresses peanut allergy in mice. Plant Biotechnol. J. 14, 1541–1550. 10.1111/pbi.12515 26801740 PMC5066676

[B27] Gruber P. Becker W. M. Hofmann T. (2005). Influence of the maillard reaction on the allergenicity of rAra h 2, a recombinant major allergen from peanut (*Arachis hypogaea*), its major epitopes, and peanut agglutinin. J. Agric. Food Chem. 53, 2289–2296. 10.1021/jf048398w 15769170

[B28] Halim A. Carlsson M. C. Madsen C. B. Brand S. Moller S. R. Olsen C. E.. (2015). Glycoproteomic analysis of seven major allergenic proteins reveals novel post-translational modifications. Mol. Cell. Proteomics 14, 191–204. 10.1074/mcp.M114.042614 25389185 PMC4288254

[B29] Hemmings O. Du Toit G. Radulovic S. Lack G. Santos A. F. (2020). Ara h 2 is the dominant peanut allergen despite similarities with Ara h 6. J. Allergy Clin. Immunol. 146, 621–630. 10.1016/j.jaci.2020.03.026 32298698 PMC7482438

[B30] Hoang J. A. Celik A. Lupinek C. Valenta R. Duan L. Dai R.. (2020). Modeling the conversion between specific IgE test platforms for nut allergens in children and adolescents. Allergy 76, 831–841. 10.1111/all.14529 32738829

[B31] Holtz B. R. Berquist B. R. Bennett L. D. Kommineni V. J. M. Munigunti R. K. White E. L.. (2015). Commercial-scale biotherapeutics manufacturing facility for plant-made pharmaceuticals. Plant Biotechnol. J. 13, 1180–1190. 10.1111/pbi.12469 26387511

[B32] Jutras P. V. Dodds I. Van Der Hoorn R. A. (2020). Proteases of *Nicotiana benthamiana*: an emerging battle for molecular farming. Curr. Opin. Biotechnol. 61, 60–65. 10.1016/j.copbio.2019.10.006 31765962

[B33] Kalic T. Kamath S. D. Ruethers T. Taki A. C. Nugraha R. Le T. T. K.. (2020). Collagen-An important fish allergen for improved diagnosis. J. Allergy Clin. Immunol. Pract. 8, 3084–3092. 10.1016/j.jaip.2020.04.063 32389794

[B34] Kalthoff D. Giritch A. Geisler K. Bettmann U. Klimyuk V. Hehnen H. R.. (2010). Immunization with plant-expressed hemagglutinin protects chickens from lethal highly pathogenic avian influenza virus H5N1 challenge infection. J. Virol. 84, 12002–12010. 10.1128/JVI.00940-10 20810729 PMC2977904

[B35] Kang I. H. Srivastava P. Ozias-Akins P. Gallo M. (2007). Temporal and spatial expression of the major allergens in developing and germinating peanut seed. Plant Physiol. 144, 836–845. 10.1104/pp.107.096933 17468222 PMC1914213

[B36] Klimyuk V. Pogue G. Herz S. Butler J. Haydon H. (2014). Production of recombinant antigens and antibodies in *Nicotiana benthamiana* using 'magnifection' technology: GMP-compliant facilities for small- and large-scale manufacturing. Plant Viral Vectors 375, 127–154. 10.1007/82_2012_212 22527176

[B37] Koppelman S. J. Vlooswijk R. A. Knippels L. M. Hessing M. Knol E. F. Van Reijsen F. C.. (2001). Quantification of major peanut allergens Ara h 1 and Ara h 2 in the peanut varieties Runner, Spanish, Virginia, and Valencia, bred in different parts of the world. Allergy 56, 132–137. 10.1034/j.1398-9995.2001.056002132.x 11167373

[B38] Krebitz M. Wagner B. Ferreira F. Peterbauer C. Campillo N. Witty M.. (2003). Plant-based heterologous expression of Mal d 2, a thaumatin-like protein and allergen of apple (*Malus domestica*), and its characterization as an antifungal protein. J. Mol. Biol. 329, 721–730. 10.1016/S0022-2836(03)00403-0 12787673

[B39] Krebitz M. Wiedermann U. Essl D. Steinkellner H. Wagner B. Turpen T. H.. (2000). Rapid production of the major birch pollen allergen Bet v 1 in *Nicotiana benthamiana* plants and its immunological *in vitro* and *in vivo* characterization. FASEB J. 14, 1279–1288. 10.1096/fasebj.14.10.1279 10877820

[B40] Krogulska A. Wood R. A. (2020). Peanut allergy diagnosis: moving from basic to more elegant testing. Pediatr. Allergy Immunol. 31, 346–357. 10.1111/pai.13215 31945225

[B41] Larsen J. M. Bang-Berthelsen C. H. Qvortrup K. Sancho A. I. Hansen A. H. Andersen K. I. H.. (2020). Production of allergen-specific immunotherapeutic agents for the treatment of food allergy. Crit. Rev. Biotechnol. 40, 881–894. 10.1080/07388551.2020.1772194 32515236

[B42] Lehmann K. Hoffmann S. Neudecker P. Suhr M. Becker W. M. Rosch P. (2003). High-yield expression in *Escherichia coli*, purification, and characterization of properly folded major peanut allergen Ara h 2. Protein Expr. Purif. 31, 250–259. 10.1016/S1046-5928(03)00190-6 14550644

[B43] Leonard R. Petersen B. O. Himly M. Kaar W. Wopfner N. Kolarich D.. (2005). Two novel types of O-glycans on the mugwort pollen allergen Art v 1 and their role in antibody binding. J. Biol. Chem. 280, 7932–7940. 10.1074/jbc.M410407200 15591314

[B44] Lew M. H. Lim R. L. (2016). Expression of a codon-optimised recombinant Ara h 2.02 peanut allergen in *Escherichia coli*. Appl. Microbiol. Biotechnol. 100, 661–671. 10.1007/s00253-015-6953-y 26411458

[B45] Li J. Shefcheck K. Callahan J. Fenselau C. (2010). Primary sequence and site-selective hydroxylation of prolines in isoforms of a major peanut allergen protein Ara h 2. Protein Sci. 19, 174–182. 10.1002/pro.295 19937656 PMC2817853

[B46] Lindbo J. A. (2007). TRBO: a high-efficiency tobacco mosaic virus RNA-based overexpression vector. Plant Physiol. 145, 1232–1240. 10.1104/pp.107.106377 17720752 PMC2151719

[B47] Lobstein J. Emrich C. A. Jeans C. Faulkner M. Riggs P. Berkmen M. (2012). SHuffle, a novel *Escherichia coli* protein expression strain capable of correctly folding disulfide bonded proteins in its cytoplasm. Microb. Cell Fact. 11:56. 10.1186/1475-2859-11-56 22569138 PMC3526497

[B48] Lomonossoff G. P. D'Aoust M. A. (2016). Plant-produced biopharmaceuticals: a case of technical developments driving clinical deployment. Science. 353, 1237–1240. 10.1126/science.aaf6638 27634524

[B49] Lowenstein H. Larsen J. N. (2001). Recombinant allergens/allergen standardization. Curr. Allergy Asthma Rep. 1, 474–479. 10.1007/s11882-001-0036-0 11892075

[B50] Lupinek C. Wollmann E. Baar A. Banerjee S. Breiteneder H. Broecker B. M.. (2014). Advances in allergen-microarray technology for diagnosis and monitoring of allergy: the MeDALL allergen-chip. Methods 66, 106–119. 10.1016/j.ymeth.2013.10.008 24161540 PMC4687054

[B51] Madsen T. D. Hansen L. H. Hintze J. Ye Z. Jebari S. Andersen D. B.. (2020). An atlas of O-linked glycosylation on peptide hormones reveals diverse biological roles. Nat. Commun. 11:4033. 10.1038/s41467-020-17473-1 32820167 PMC7441158

[B52] Mair A. Pedrotti L. Wurzinger B. Anrather D. Simeunovic A. Weiste C.. (2015). SnRK1-triggered switch of bZIP63 dimerization mediates the low-energy response in plants. Elife 4:e05828. 10.7554/eLife.05828 26263501 PMC4558565

[B53] Marillonnet S. Giritch A. Gils M. Kandzia R. Klimyuk V. Gleba Y. (2004). In planta engineering of viral RNA replicons: efficient assembly by recombination of DNA modules delivered by Agrobacterium. Proc. Natl. Acad. Sci. U.S.A. 101, 6852–6857. 10.1073/pnas.0400149101 15103020 PMC406431

[B54] Marillonnet S. Thoeringer C. Kandzia R. Klimyuk V. Gleba Y. (2005). Systemic *Agrobacterium tumefaciens*-mediated transfection of viral replicons for efficient transient expression in plants. Nat. Biotechnol. 23, 718–723. 10.1038/nbt1094 15883585

[B55] Mathieu-Rivet E. Mati-Baouche N. Walet-Balieu M. L. Lerouge P. Bardor M. (2020). N- and O-Glycosylation pathways in the microalgae polyphyletic group. Front. Plant Sci. 11:609993. 10.3389/fpls.2020.609993 33391324 PMC7773692

[B56] Mócsai R. Göritzer K. Stenitzer D. Maresch D. Strasser R. Altmann F. (2021). Prolyl hydroxylase paralogs in *Nicotiana benthamiana* show high similarity with regard to substrate specificity. Front. Plant Sci. 12:636597. 10.3389/fpls.2021.636597 33737944 PMC7960765

[B57] Munoz-Talavera A. Gomez-Lim M. A. Salazar-Olivo L. A. Reinders J. Lim K. Escobedo-Moratilla A.. (2019). Expression of the biologically active insulin analog SCI-57 in *Nicotiana benthamiana*. Front. Pharmacol. 10:1335. 10.3389/fphar.2019.01335 31798448 PMC6868099

[B58] Nakamura R. Uchida Y. Higuchi M. Nakamura R. Tsuge I. Urisu A.. (2010). A convenient and sensitive allergy test: IgE crosslinking-induced luciferase expression in cultured mast cells. Allergy 65, 1266–1273. 10.1111/j.1398-9995.2010.02363.x 20374229 PMC3066406

[B59] Ozias-Akins P. Breiteneder H. (2019). The functional biology of peanut allergens and possible links to their allergenicity. Allergy 74, 888–898. 10.1111/all.13719 30636003 PMC6563476

[B60] Palladino C. Narzt M. S. Bublin M. Schreiner M. Humeniuk P. Gschwandtner M.. (2018). Peanut lipids display potential adjuvanticity by triggering a pro-inflammatory response in human keratinocytes. Allergy 73, 1746–1749. 10.1111/all.13475 29747215 PMC6095042

[B61] Palmer G. W. Dibbern D. A. Jr Burks A. W. Bannon G. A. Bock S. A. Porterfield H. S.. (2005). Comparative potency of Ara h 1 and Ara h 2 in immunochemical and functional assays of allergenicity. Clin. Immunol. 115, 302–312. 10.1016/j.clim.2005.02.011 15893698

[B62] Park K. H. Lee J. Sim D. W. Lee S. C. (2018). Comparison of singleplex specific IgE detection immunoassays: immunoCAP Phadia 250 and immulite 2000 3gAllergy. Ann. Lab. Med. 38, 23–31. 10.3343/alm.2018.38.1.23 29071815 PMC5700143

[B63] Park Y. Min K. Kim N. H. Kim J. H. Park M. Kang H.. (2021). Porcine circovirus 2 capsid protein produced in *N. benthamiana* forms virus-like particles that elicit production of virus-neutralizing antibodies in guinea pigs. Nat. Biotechnol. 63, 29–36. 10.1016/j.nbt.2021.02.005 33667631

[B64] Perez-Riverol Y. Csordas A. Bai J. Bernal-Llinares M. Hewapathirana S. Kundu D. J.. (2019). The PRIDE database and related tools and resources in 2019: improving support for quantification data. Nucleic Acids Res. 47, D442–D450. 10.1093/nar/gky1106 30395289 PMC6323896

[B65] Petersen A. Schramm G. Schlaak M. Becker W. M. (1998). Post-translational modifications influence IgE reactivity to the major allergen Phl p 1 of timothy grass pollen. Clin. Exp. Allergy 28, 315–321. 10.1046/j.1365-2222.1998.00221.x 9543081

[B66] Petersen B. L. Macalister C. A. Ulvskov P. (2021). Plant protein O-arabinosylation. Front. Plant Sci. 12:645219. 10.3389/fpls.2021.645219 33815452 PMC8012813

[B67] Platts-Mills T. A. (2015). The allergy epidemics: 1870-2010. J. Allergy Clin. Immunol. 136, 3–13. 10.1016/j.jaci.2015.03.048 26145982 PMC4617537

[B68] Radauer C. Bublin M. Wagner S. Mari A. Breiteneder H. (2008). Allergens are distributed into few protein families and possess a restricted number of biochemical functions. J. Allergy Clin. Immunol. 121, 847–852. 10.1016/j.jaci.2008.01.025 18395549

[B69] Reynolds L. A. Finlay B. B. (2017). Early life factors that affect allergy development. Nat. Rev. Immunol. 17, 518–528. 10.1038/nri.2017.39 28504257

[B70] Sancho A. I. Hoffmann-Sommergruber K. Alessandri S. Conti A. Giuffrida M. G. Shewry P.. (2010). Authentication of food allergen quality by physicochemical and immunological methods. Clin. Exp. Allergy 40, 973–986. 10.1111/j.1365-2222.2010.03534.x 20642576

[B71] Santoni M. Ciardiello M. A. Zampieri R. Pezzotti M. Giangrieco I. Rafaiani C.. (2019). Plant-made Bet v 1 for molecular diagnosis. Front. Plant Sci. 10:1273. 10.3389/fpls.2019.01273 31649716 PMC6795700

[B72] Santos A. F. Barbosa-Morais N. L. Hurlburt B. K. Ramaswamy S. Hemmings O. Kwok M.. (2020). IgE to epitopes of Ara h 2 enhance the diagnostic accuracy of Ara h 2-specific IgE. Allergy 75, 2309–2318. 10.1111/all.14301 32248566

[B73] Schillberg S. Raven N. Spiegel H. Rasche S. Buntru M. (2019). Critical analysis of the commercial potential of plants for the production of recombinant proteins. Front. Plant Sci. 10:720. 10.3389/fpls.2019.00720 31244868 PMC6579924

[B74] Schmid-Grendelmeier P. Holzmann D. Himly M. Weichel M. Tresch S. Ruckert B.. (2003). Native Art v 1 and recombinant Art v 1 are able to induce humoral and T cell-mediated in vitro and in vivo responses in mugwort allergy. J. Allergy Clin. Immunol. 111, 1328–1336. 10.1067/mai.2003.1495 12789236

[B75] Schmidt G. Gadermaier G. Pertl H. Siegert M. Oksman-Caldentey K. M. Ritala A.. (2008). Production of recombinant allergens in plants. Phytochem. Rev. 7, 539–552. 10.1007/s11101-008-9099-z 21258627 PMC3024541

[B76] Schneider C. A. Rasband W. S. Eliceiri K. W. (2012). NIH Image to ImageJ: 25 years of image analysis. Nat. Methods 9, 671–675. 10.1038/nmeth.2089 22930834 PMC5554542

[B77] Schoberer J. Shin Y.-J. Vavra U. Veit C. Strasser R. (2018). Analysis of protein glycosylation in the ER. Methods Mol. Biol. 1691, 205–222. 10.1007/978-1-4939-7389-7_16 29043680 PMC7039702

[B78] Shpak E. Leykam J. F. Kieliszewski M. J. (1999). Synthetic genes for glycoprotein design and the elucidation of hydroxyproline-O-glycosylation codes. Proc. Natl. Acad. Sci. U.S.A. 96, 14736–14741. 10.1073/pnas.96.26.14736 10611282 PMC24717

[B79] Slater A. Scott N. Fowler M. (2008). Molecular farming, in Plant Biotechnology: The Genetic Manipulation of Plants (Oxford University Press Inc.), 267–315.

[B80] Stanley J. S. King N. Burks A. W. Huang S. K. Sampson H. Cockrell G.. (1997). Identification and mutational analysis of the immunodominant IgE binding epitopes of the major peanut allergen Ara h 2. Arch. Biochem. Biophys. 342, 244–253. 10.1006/abbi.1997.9998 9186485

[B81] Strasser R. (2016). Plant protein glycosylation. Glycobiology 26, 926–939. 10.1093/glycob/cww023 26911286 PMC5045529

[B82] Tscheppe A. Palmberger D. Van Rijt L. Kalic T. Mayr V. Palladino C.. (2020). Development of a novel Ara h 2 hypoallergen with no IgE binding or anaphylactogenic activity. J. Allergy Clin. Immunol. 145, 229–238. 10.1016/j.jaci.2019.08.036 31525384 PMC7100897

[B83] Tusé D. Tu T. Mcdonald K. A. (2014). Manufacturing economics of plant-made biologics: case studies in therapeutic and industrial enzymes. Biomed Res. Int. 2014:256135. 10.1155/2014/256135 24977145 PMC4058100

[B84] Ueberham E. Spiegel H. Havenith H. Rautenberger P. Lidzba N. Schillberg S.. (2019). Simplified tracking of a soy allergen in processed food using a monoclonal antibody-based sandwich ELISA targeting the soybean 2S albumin Gly m 8. J. Agric. Food Chem. 67, 8660–8667. 10.1021/acs.jafc.9b02717 31298531

[B85] Üzülmez Ö. Kalic T. Breiteneder H. (2020). Advances and novel developments in molecular allergology. Allergy 75, 3027–3038. 10.1111/all.14579 32882057 PMC7756543

[B86] Valenta R. Karaulov A. Niederberger V. Gattinger P. Van Hage M. Flicker S.. (2018a). Molecular aspects of allergens and allergy. Adv. Immunol. 138, 195–256. 10.1016/bs.ai.2018.03.002 29731005

[B87] Valenta R. Karaulov A. Niederberger V. Zhernov Y. Elisyutina O. Campana R.. (2018b). Allergen extracts for *in vivo* diagnosis and treatment of allergy: is there a future? J. Allergy Clin. Immunol. Pract. 6, 1845–1855. 10.1016/j.jaip.2018.08.032 30297269 PMC6390933

[B88] Vissers Y. M. Iwan M. Adel-Patient K. Stahl Skov P. Rigby N. M. Johnson P. E.. (2011). Effect of roasting on the allergenicity of major peanut allergens Ara h 1 and Ara h 2/6: the necessity of degranulation assays. Clin. Exp. Allergy 41, 1631–1642. 10.1111/j.1365-2222.2011.03830.x 21801247

[B89] Way M. Pope B. Gooch J. Hawkins M. Weeds A. G. (1990). Identification of a region in segment 1 of gelsolin critical for actin binding. EMBO J. 9, 4103–4109. 10.1002/j.1460-2075.1990.tb07632.x 2174356 PMC552183

[B90] Yamada Y. Kidoguchi M. Yata A. Nakamura T. Yoshida H. Kato Y.. (2020). High-yield production of the major birch pollen allergen Bet v 1 with allergen immunogenicity in *Nicotiana benthamiana*. Front. Plant Sci. 11:344. 10.3389/fpls.2020.00344 32300351 PMC7142267

[B91] Yamamoto T. Hoshikawa K. Ezura K. Okazawa R. Fujita S. Takaoka M.. (2018). Improvement of the transient expression system for production of recombinant proteins in plants. Sci. Rep. 8:4755. 10.1038/s41598-018-23024-y 29555968 PMC5859073

[B92] Yang S. J. Carter S. A. Cole A. B. Cheng N. H. Nelson R. S. (2004). A natural variant of a host RNA-dependent RNA polymerase is associated with increased susceptibility to viruses by *Nicotiana benthamiana*. Proc. Natl. Acad. Sci. U.S.A. 101, 6297–6302. 10.1073/pnas.0304346101 15079073 PMC395963

[B93] Youl J. J. Bacic A. Oxley D. (1998). Arabinogalactan-proteins from Nicotiana alata and Pyrus communis contain glycosylphosphatidylinositol membrane anchors. Proc. Natl. Acad. Sci. U.S.A. 95, 7921–7926. 10.1073/pnas.95.14.7921 9653116 PMC20905

[B94] Yuasa K. Toyooka K. Fukuda H. Matsuoka K. (2005). Membrane-anchored prolyl hydroxylase with an export signal from the endoplasmic reticulum. Plant J. 41, 81–94. 10.1111/j.1365-313X.2004.02279.x 15610351

[B95] Zhuang Y. Dreskin S. C. (2013). Redefining the major peanut allergens. Immunol. Res. 55, 125–134. 10.1007/s12026-012-8355-x 22948807 PMC4451826

