# Supplementary figures and images for: The Major Peanut Allergen Ara h 2 Produced in Nicotiana benthamiana Contains Hydroxyprolines and Is a Viable Alternative to the E. Coli Product in Allergy Diagnosis
Source: Front Plant Sci. 2021 Oct 4;12:723363. doi: 10.3389/fpls.2021.723363 (PMC8522509; doi:10.3389/fpls.2021.723363)

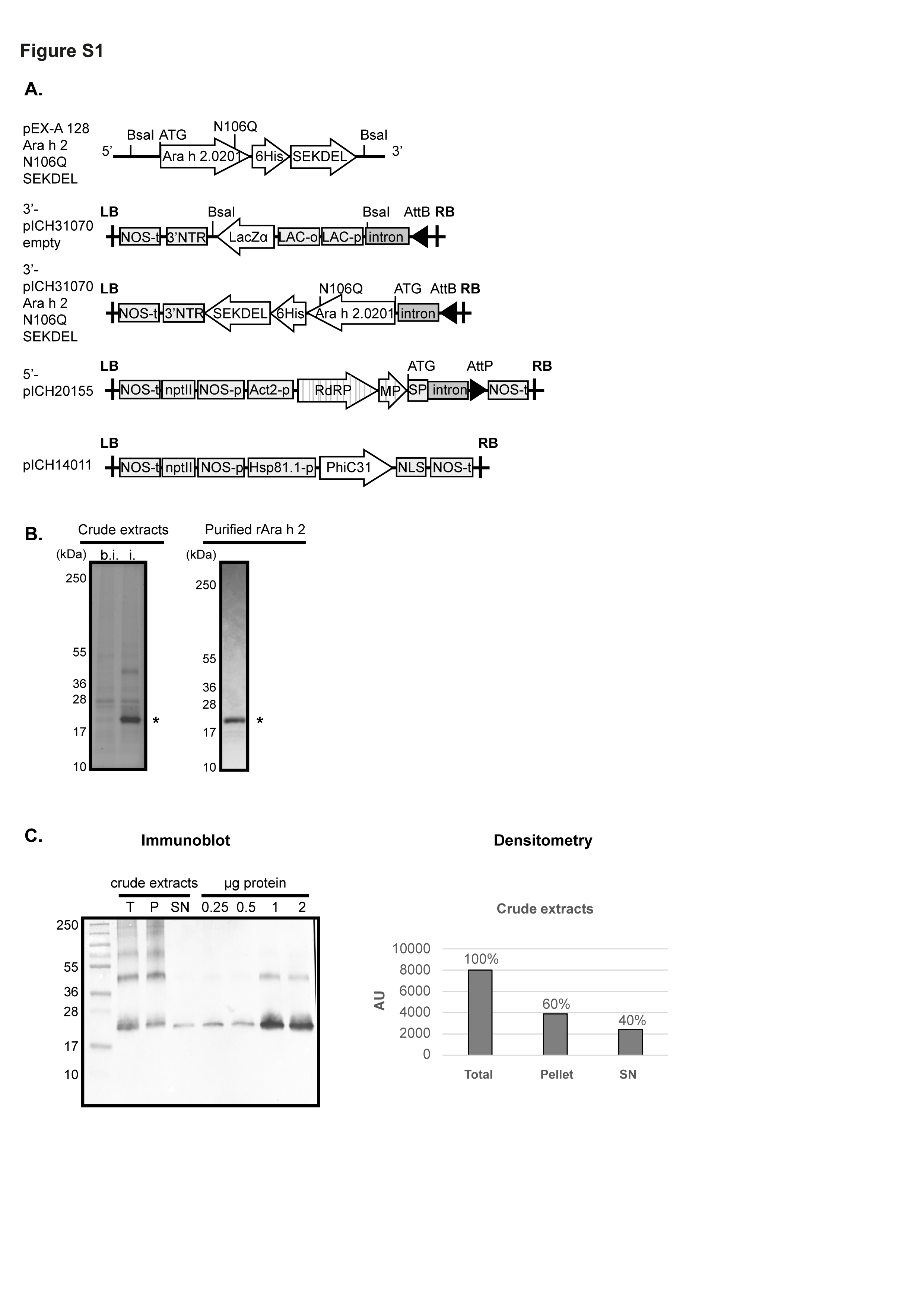

Supplement: Supplementary file 1 [file Image_1.TIF]

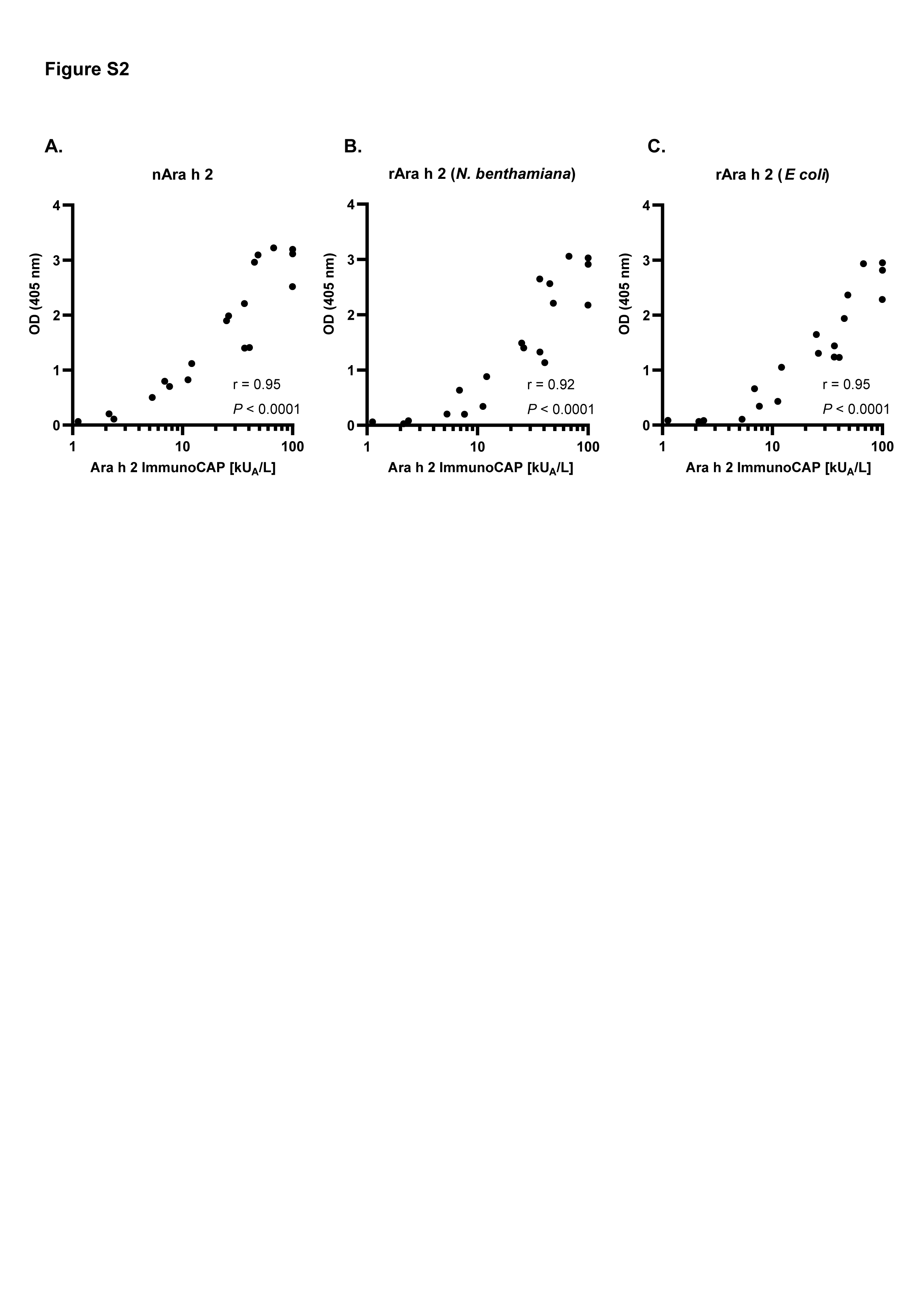

Supplement: Supplementary file 2 [file Image_2.TIF]

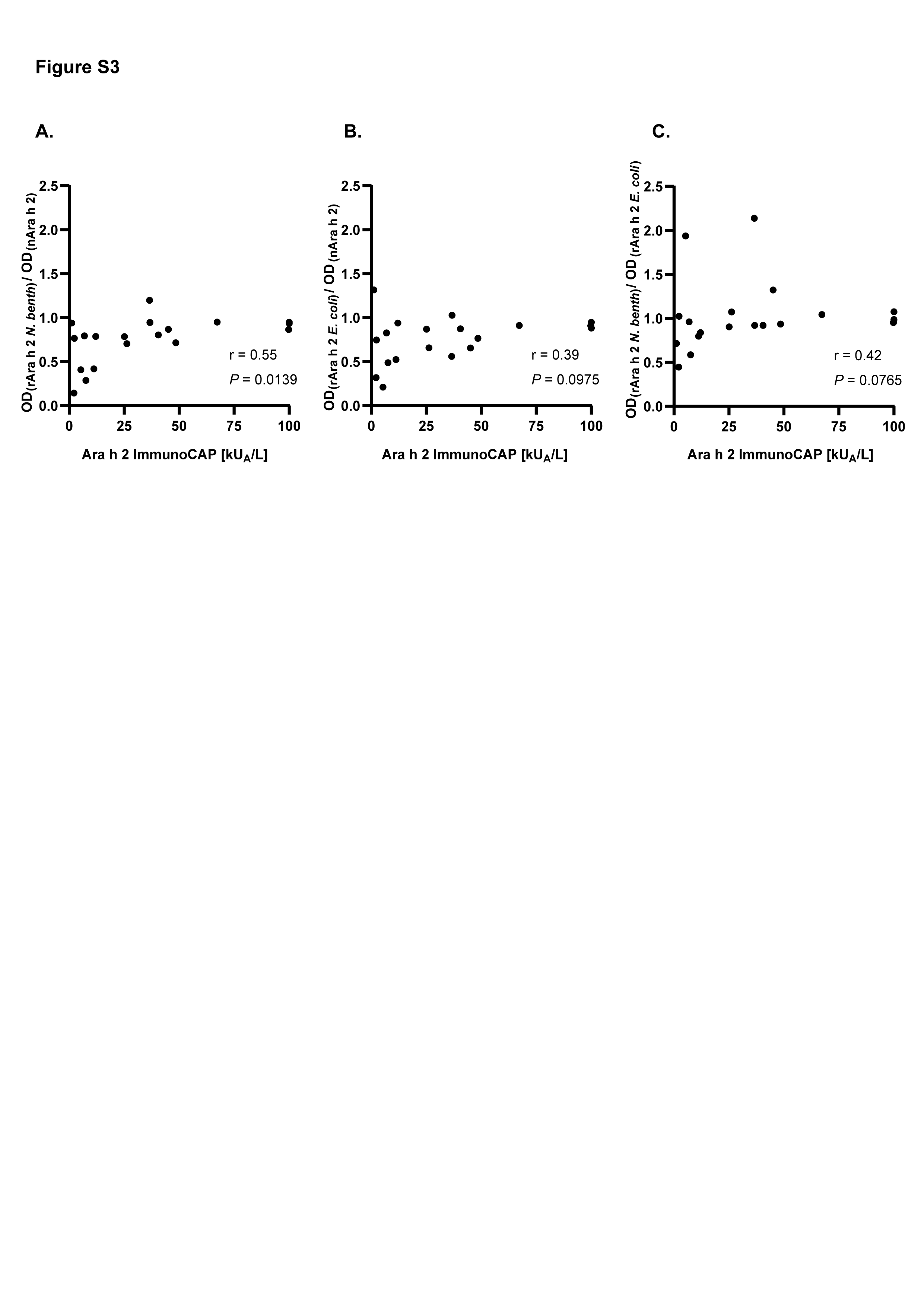

Supplement: Supplementary file 3 [file Image_3.TIF]

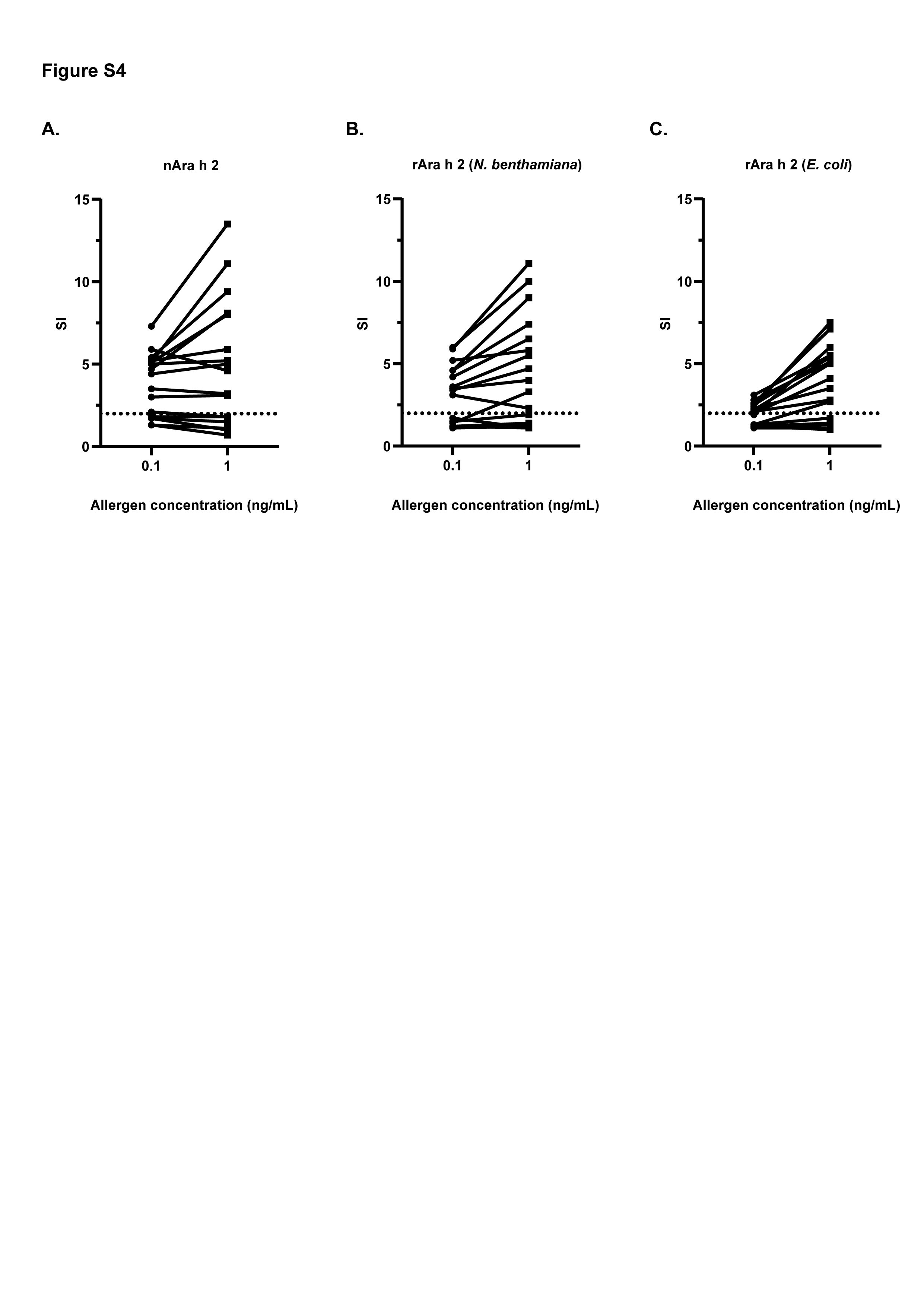

Supplement: Supplementary file 4 [file Image_4.TIF]

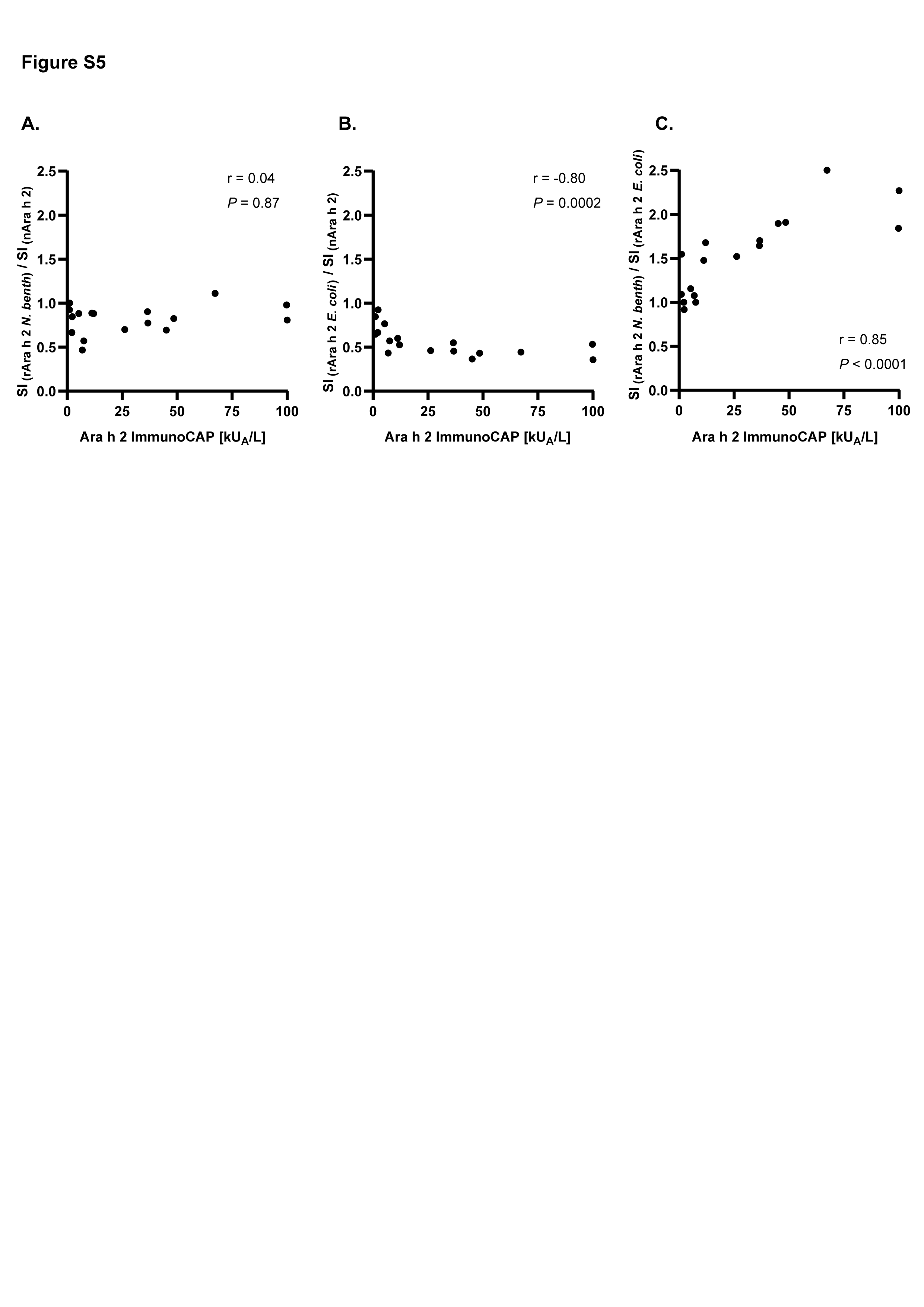

Supplement: Supplementary file 5 [file Image_5.TIF]
