# Supplementary material for: The Major Peanut Allergen Ara h 2 Produced in Nicotiana benthamiana Contains Hydroxyprolines and Is a Viable Alternative to the E. Coli Product in Allergy Diagnosis
Source: Front Plant Sci. 2021 Oct 4;12:723363. doi: 10.3389/fpls.2021.723363 (PMC8522509; doi:10.3389/fpls.2021.723363)
Supplement: Supplementary file 7 [file Data_Sheet_2.docx]

Supplementary Material to “The major peanut allergen Ara h 2 produced in Nicotiana benthamiana contains hydroxyprolines and is a viable alternative to the E. coli product in allergy diagnosis”

# Supplementary table and table legend:

Table S1. Patients’ characteristics. AN: anaphylaxis grade according to Ring and Messmer (Ring and Messmer, 1977), n.d.: not done, OAS: oral allergy syndrome, GI: gastrointestinal

| Peanut allergic patients | Sex | Age (yrs) | Total IgE (kU/L) | Specific IgE (kU/L) | | | | | | Clinical Symptoms | Skin prick test with peanut extract (mm) |
| --- | --- | --- | --- | --- | --- | --- | --- | --- | --- | --- | --- |
|  |  |  |  | **Peanut** | **Ara h 1** | **Ara h 2** | **Ara h 3** | **Ara h 8** | **Ara h 9** |  |  |
| P1 | m | 29 | 640 | >100 | 64.10 | 99.80 | 22.00 | n.d. | n.d. | AN Grade 3 | 13 |
| P2 | f | 21 | 472 | 93.80 | 24.50 | 48.50 | 10.70 | 25.90 | n.d. | AN Grade 1 | 4 |
| P3 | f | 23 | 297 | 80.10 | 66.70 | 40.50 | 8.60 | 1.67 | n.d. | AN Grade 1 | n.d. |
| P4 | m | 47 | 126 | n.d. | n.d. | 25.10 | n.d. | n.d. | n.d. | AN Grade 3 | 9 |
| P5 | f | 37 | 430 | n.d. | 53.70 | 36.70 | 25.70 | n.d. | n.d. | AN Grade 1 | n.d. |
| P6 | f | 34 | 205 | 4.14 | 0.08 | 2.37 | 0.18 | 0.84 | n.d. | OAS | 4 |
| P7 | f | 31 | 428 | n.d. | 54.10 | >100 | 17.10 | 0.01 | 0.02 | AN Grade 3 | 7 |
| P8 | m | 40 | 87 | n.d. | 4.24 | 5.30 | n.d. | n.d. | n.d. | AN Grade 3 | 9 |
| P9 | m | 22 | 578 | 99.30 | n.d. | 67.30 | n.d. | n.d. | n.d. | OAS, AN Grade 2 | 12 |
| P10 | f | 46 | 1,636 | 38.80 | n.d. | 11.20 | n.d. | n.d. | n.d. | AN Grade 4 | 3 |
| P11 | m | 54 | 1,225 | n.d. | 0.03 | 1.10 | 0.04 | n.d. | 0.29 | AN Grade 4 | 3 |
| P12 | f | 45 | 929 | n.d. | 0.06 | 1.12 | 0.00 | 1.32 | 0.20 | AN Grade 1 | 6 |
| P13 | f | 44 | 78 | n.d. | 0.64 | 2.14 | 0.03 | n.d. | 0.00 | AN Grade 2 | 16 |
| P14 | f | 35 | 506 | 25.50 | n.d. | 6.87 | n.d. | n.d. | n.d. | AN Grade 1 | 7 |
| P15 | f | 30 | 362 | n.d. | 4.48 | 7.62 | 0.01 | 3.45 | 0.01 | AN Grade 2 | n.d. |
| P16 | m | 46 | 125 | n.d. | 21.40 | 12.10 | 6.60 | 0.16 | 0.00 | AN Grade 1 | 10 |
| P17 | m | 36 | 257 | n.d. | n.d. | 26.20 | n.d. | n.d. | n.d. | OAS | 10 |
| P18 | f | 28 | 716 | n.d. | 41.90 | 45.10 | 7.00 | n.d. | n.d. | AN Grade 2 | 8 |
| P19 | f | 22 | 680 | n.d. | >100 | >100 | n.d. | 0.03 | 0.03 | AN Grade 1 | 11 |
| P20 | f | 18 | 383 | n.d. | 27.90 | 36.50 | 11.60 | n.d. | n.d. | AN Grade 2 | 9 |
|  | | | | | | | | | | | |
| Non-allergic healthy control sera | **Sex** | **Age (yrs)** | |  | | | | | | | |
| P21 | f | 45 | |  | | | | | | | |
| P22 | m | 27 | |  | | | | | | | |
| P23 | f | 43 | |  | | | | | | | |
|  | | | | | | | | | | | |
| Non-peanut allergic control sera | **Sex** | **Age (yrs)** | | **Total IgE (kU/L)** | | **Allergy** | | | |  | |
| P24 | m | 41 | | 94.30 | | Timothy grass | | | |  | |
| P25 | m | 42 | | n.d. | | Birch pollen, apple | | | |  | |
| P26 | f | 49 | | n.d. | | Birch pollen | | | |  | |

# Supplementary figure legends:

**Supplementary Figure S1.** Design of plant expression vectors and Agrobacterium-mediated transient expression of heterologous proteins in *N. benthamiana*. **(A)** The Ara h 2 coding sequence with a C-terminal hexa-histidine tag followed by the ER-retention signal (SEKDEL) amplified in pEX-A 128 was inserted into the magnICON^®^ 3’-pICH31070 provector creating 3’-pICH31070 Ara h 2 N106Q SEKDEL. The 5’-provector pICH20155 carrying viral genes was used in combination with the 3’-provector. The pICH14011 encoded the PhiC31 integrase. **(B)** Presence of rAra h 2 in crude protein extracts of infiltrated leaves (i.) and buffer infiltrated leaves as controls (b.i.). *N. benthamiana*-produced affinity purified rAra h 2 migrated as a single band as marked on SDS-PAGE. **(C)** Relative quantification of rAra h 2 in the total leaf homogenates (T) as well as in the pellet (P) and the supernatant (SN) after centrifugation. Equimolar amounts corresponding to 9 µg protein were loaded per lane for the T, P, SN fractions of homogenized leaf suspensions. Purified rAra h 2 loaded from 0.25 µ to 2 µg were separated by SDS-PAGE. Membrane transferred proteins were detected by a monoclonal anti-Ara h 2 antibody and quantified by densitometry using ImageJ 1.8.0 (Schneider et al., 2012). Percentages above the graph bars correspond to approximate values calculated by comparing with the standard curve obtained with purified Ara h 2.

**Supplementary Figure S2.** The IgE ELISA results for **(A)** nAra h 2, **(B)** rAra h 2 (*N. benthamiana*), **(C)** rAra h 2 (*E. coli*) were correlated with patients’ Ara h 2-sIgE values measured by ImmunoCAP.

**Supplementary Figure S3.** **(A)** The ratio of IgE-binding to rAra h 2 (*N. benthamiana*) and to nAra h 2 was correlated with patients’ ImmunoCAP values. **(B)** The ratio of IgE-binding to rAra h 2 (*E. coli*) and to nAra h 2 was correlated with patients’ ImmunoCAP values. **(C)** The ratio of IgE-binding to rAra h 2 (*N. benthamiana*) and to rAra h 2 (*E. coli*) was correlated with patients’ ImmunoCAP values.

**Supplementary Figure S4. (A)** Patient-specific basophil activation induced by nAra h 2 is shown at both concentrations (0.1 ng/mL and 1 ng/mL) used in the assay. **(B)** Shown for rAra h 2 (*N. benthamiana*) as in A. **(C)** Shown for rAra h 2 (*E. coli*) as in A.

**Supplementary Figure S5. (A)** The ratio of SI from RBL assays at 0.1 ng/mL for rAra h 2 (*N. benthamiana*) and nAra h 2 was correlated with patients’ ImmunoCAP values. **(B)** The ratio of SI from RBL assays at 0.1 ng/mL for rAra h 2 (*E. coli*) and nAra h 2 was correlated with patients’ ImmunoCAP values. **(C)** The ratio of SI from RBL assays at 0.1 ng/mL for rAra h 2 (*N. benthamiana*) and rAra h 2 (*E. coli*) was correlated with patients’ ImmunoCAP values.

# Supplementary Materials to Figure S1B


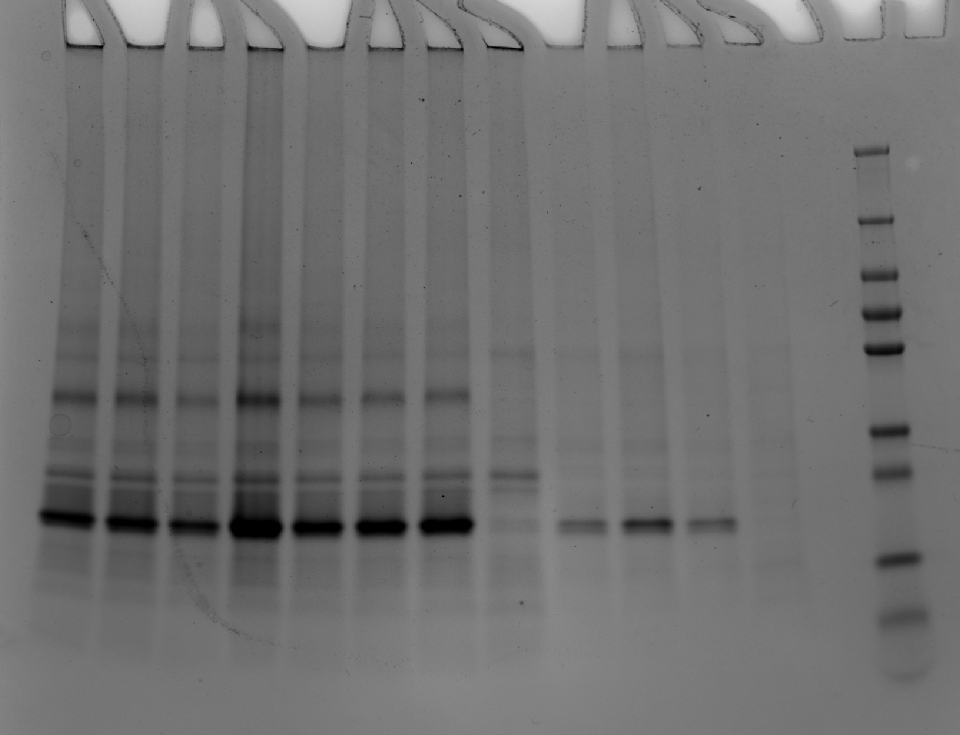

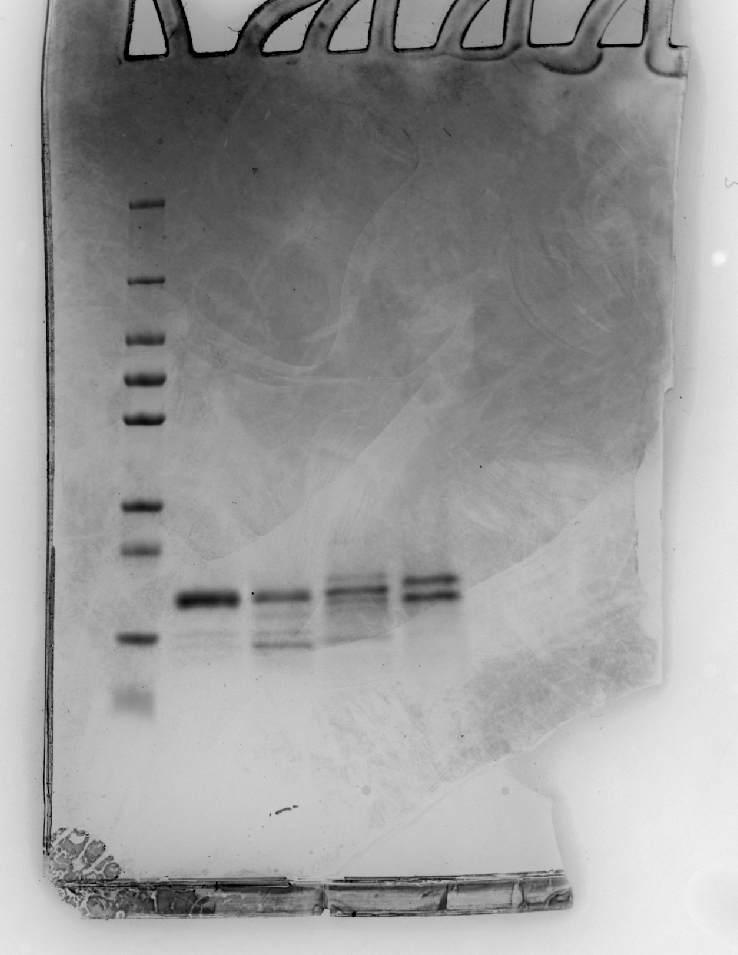


# Supplementary References

Schneider, C.A., Rasband, W.S., and Eliceiri, K.W. (2012). NIH Image to ImageJ: 25 years of image analysis. *Nat Methods* 9, 671-675.

Ring, J., and Messmer, K. (1977). Incidence and severity of anaphylactoid reactions to colloid volume substitutes. *Lancet* 1**,** 466-469.

**
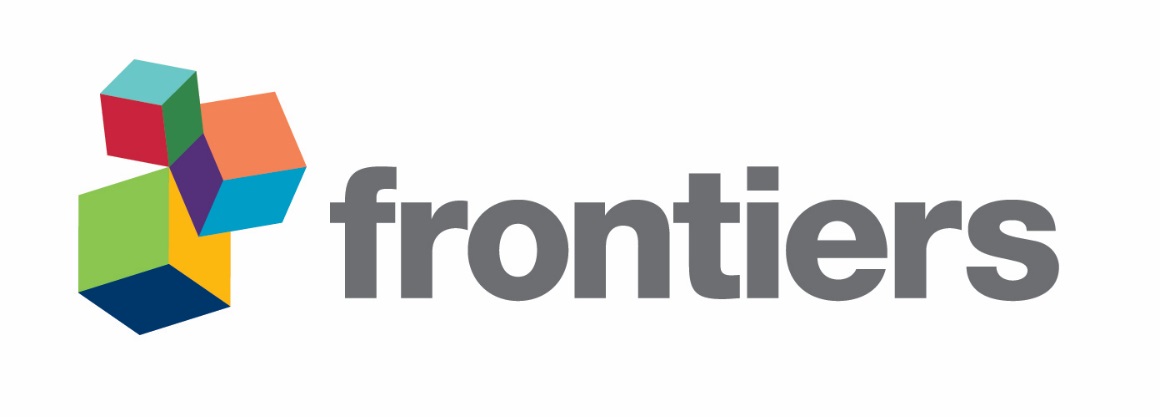
**
